# Salivary Extracellular Vesicle Proteome as a Molecular Bridge Between Periodontitis and Neurodegeneration: Mechanisms, Biomarkers, and Therapeutic Opportunities

**DOI:** 10.1080/17590914.2026.2736964

**Published:** 2026-10-06

**Authors:** Md. Azhar, Rishabha Malviya, Vandana, Phool Chandra, Sathvik Belagodu Sridhar, Javedh Shareef, Tarun Wadhwa, Daniel Arockiam

**Affiliations:** a Department of Pharmacy, School of Medical and Allied Sciences, Galgotias University, Greater Noida, Uttar Pradesh, India; b School of Pharmacy, Sharda University, Greater Noida, Uttar Pradesh, India; c Teerthanker Mahaveer College of Pharmacy, Teerthanker Mahaveer University, Moradabad, Uttar Pradesh, India; d College of Pharmacy, Dubai Medical University, Dubai, UAE; e RAK College of Pharmacy, RAK Medical & Health Sciences University, Ras Al Khaimah, UAE; f School of Engineering and IT, Manipal Academy of Higher Education, Dubai, UAE

**Keywords:** Biomarkers, EV proteomics, extracellular vesicle cargo, neurodegeneration, neuroinflammation, periodontitis, salivary extracellular vesicles

## Abstract

Periodontitis is a chronic inflammatory condition associated with systemic inflammation and an increased risk of neurodegenerative disorders. Salivary extracellular vesicles (EVs) carry molecular cargo in an enclosed form and could serve as a non-invasive platform to explore communication between the oral system and the brain. The review analyzes the current evidence on salivary EV proteomes, their mechanistic links to neurodegeneration, and their biomarker potential. This is a narrative review, and PRISMA-2020 reporting was used as a transparency tool only. The following keywords were used to search the databases PubMed/MEDLINE, Scopus, and Web of Science for publications from 2009 to 2026: periodontal disease, periodontitis, salivary EVs, proteomics, neuroinflammation, blood-brain barrier (BBB) dysfunction, and neurodegeneration. After applying the structured screening and eligibility assessment procedure, 112 studies were included in the qualitative synthesis. Synthesized evidence suggests that cytokines, complement proteins, proteases, acute-phase mediators, and oxidative-stress signals are produced during periodontal inflammation and could be detected in salivary EV cargo. These molecular signals converge on TLR2/TLR4-NF-κB, the NLRP3 inflammasome, HMGB1-RAGE, complement C3/C1q, MMP-9-mediated BBB disruption, IL-1β/IL-6/TNF-α signaling, and oxidative stress-mitochondrial injury, potentially leading to endothelial dysfunction, BBB permeability, microglial activation, and neuronal injury. The results indicate that salivary EVs are not only biomarkers but are biologically active, suggesting a possible molecular basis for the link between periodontal inflammation and neurodegenerative processes. This review incorporates the latest data related to salivary EV proteomics, periodontal inflammation, BBB dysfunction, neuroimmune signaling, and biomarker development, with a focus on EV engineering and other emerging strategies as potential avenues for future diagnosis and therapy.

## GRAPHICAL ABSTRACT

**Figure:**
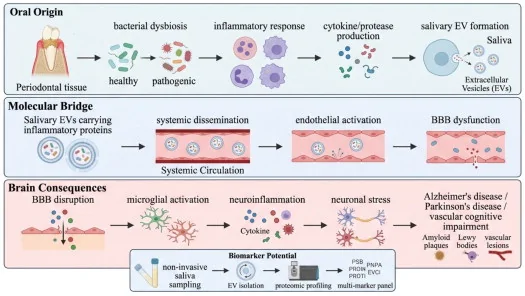

## Introduction

Neurodegenerative disorders and periodontitis may share some inflammatory and immune-related pathways. Periodontitis is a common chronic disease of the supporting tissues of the teeth that, over time, causes pocket formation, breakdown of the supporting structure of the teeth, and bone loss. Neurodegenerative conditions, such as Alzheimer’s disease and Parkinson’s disease, pose a significant burden to patients, families, and health systems due to the slow progression of the disease, its impact on a person’s ability to function, and the requirement for long-term care. Their relationship is significant, as oral inflammation may affect the whole body. Continued periodontal damage may contribute to immune stress, which can contribute to a less stable biological environment for the aging brain. The clinical implications of this association are significant as both conditions are common, chronic, and linked to progressive tissue or neurological damage (X. Li et al., 2022; R. P. H. Wang et al., 2019).

Saliva is a convenient biofluid for translational research. Collection is non-invasive, quick, repeatable, and does not subject the individual to discomfort or expense associated with blood collection. Saliva also mirrors a wide variety of biological information. It is rich in proteins, enzymes, immune mediators, nucleic acids, microbial products, and vesicle-associated cargo, thus reflecting both local oral disturbances and overall body signals. Saliva is therefore a useful screening, monitoring, and longitudinal follow-up tool. Advances in salivary diagnostic research have been aided by proteomics, genomics, metabolomics, and EV-based analysis (Amado et al., 2013; Constantin et al., 2025; Giannobile et al., 2009).

Extracellular vesicles (EVs) in saliva are the most informative carrier system. These vesicles prevent rapid degradation of their contents and help to ensure the stability of proteins and other molecular signals more effectively than many free soluble molecules. Salivary EVs can carry membrane proteins, inflammatory mediators, immune regulators, and disease-linked cargo that can indicate the state of the oral tissues. Vesicle-based packaging matters because it supports intercellular communication and enhances signal stability during transit within the oral environment and circulation. Recent reviews highlight that salivary EVs are not inert particles. They are biologically active, diagnostically useful, and have translational potential, particularly in relation to oral diseases and other systemic diseases (Chiabotto et al., 2019; Wu et al., 2023).

This review is based on the concept that the inflammatory process of periodontitis can produce a molecular load found in salivary EVs. Pathogen-related signals, complement factors, proteases, oxidative stress markers, and host inflammatory proteins can cross the periodontal lesion and enter larger body compartments. Thereafter, it may contribute to endothelial cell stress, impairment of the blood-brain barrier, and a pro-inflammatory brain environment. Experimental and clinical evidence already exists for a correlation between chronic periodontitis, cognitive impairments, and neuroinflammation, which may be relevant to Alzheimer-type pathology and to a more general neurodegenerative process (X. Li et al., 2022; R. P. H. Wang et al., 2019). Importantly, the proposed relationship is not limited to microbial factors. The host response itself may carry the strongest signal, and EVs provide a strong platform for capturing a protected, measurable signal.

This review focuses on the salivary EV proteome. The aim is not to retell oral microbiology alone, and not to repeat broad saliva biomarker discussions generically. The main question is molecular bridge-building: The pathway from periodontal inflammation to salivary EV release, systemic spread, blood-brain barrier stress, microglial activation, and neurodegenerative risk. Such a focus gives the review a more translational focus. This also opens the way for the development of biomarkers, disease stratification, and EV engineering for future directions. This is useful in the practical aspect. Beyond general inflammation summaries, a proteome-based EV model can generate more robust biomarker panels, aid clinical interpretation of data, and provide more accurate oral-brain risk mapping (Chiabotto et al., 2019; Cui et al., 2024; Wu et al., 2023). Taken together, the literature provides a basis for exploring the possibility of molecular communication between periodontal inflammation and neurologic processes via EVs found in saliva. However, direct evidence for the continual transport of EVs from periodontal tissue into the CNS remains insufficient. Thus, this review proposes the oral-brain relationship as a framework compatible with existing mechanistic, proteomic, and biomarker data and offering new opportunities for future translational research (Dhingra et al., 2026; Giannobile et al., 2009). The suggested oral-brain axis is a working hypothesis describing potential interactions among periodontal inflammation, salivary EV changes, systemic inflammatory signaling, BBB dysfunction, and neuroinflammatory responses, as shown in Figure 1. The order of these events has not yet been directly proven by experiment or by longitudinal clinical studies.

**Figure 1.**
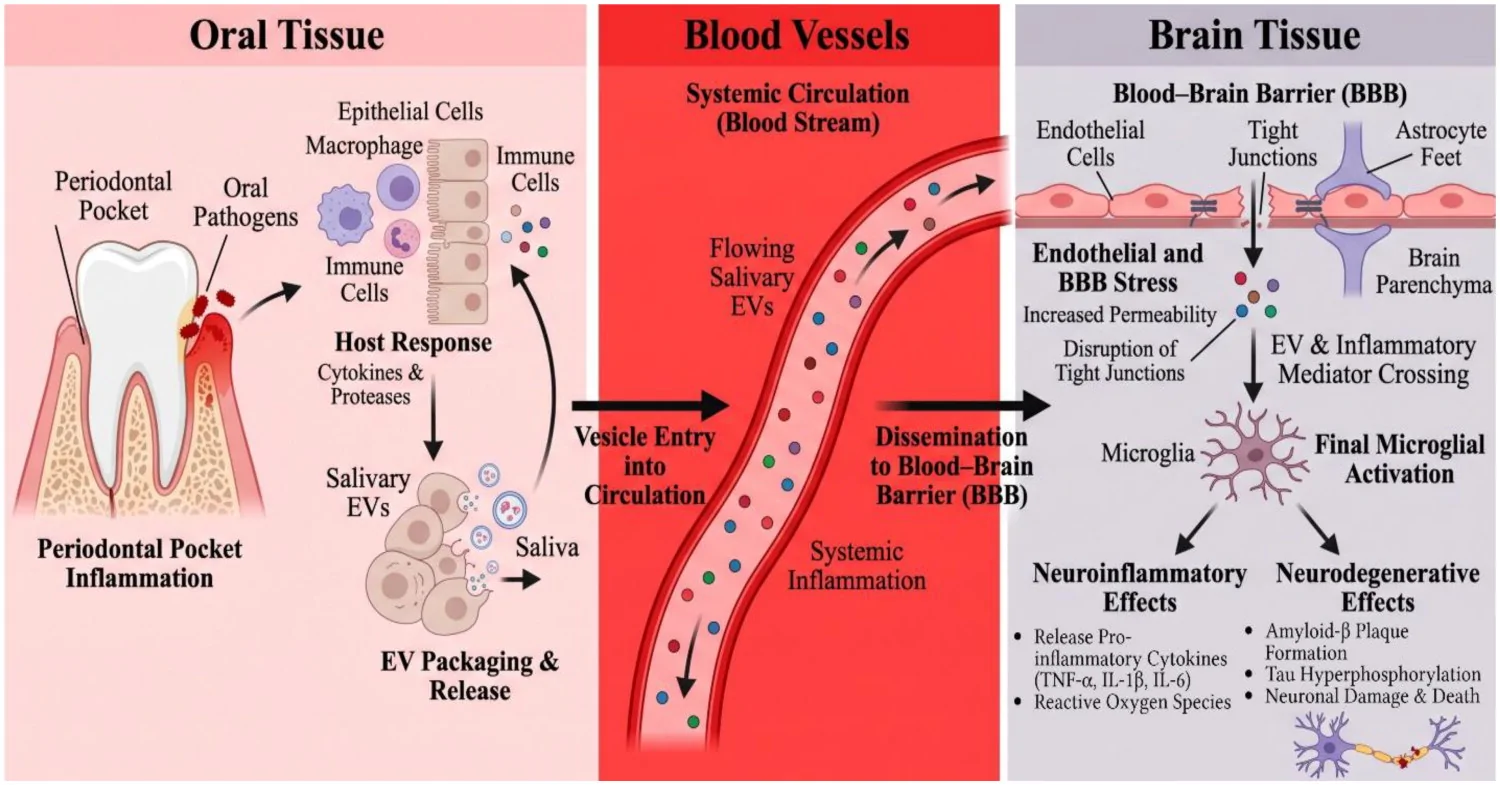
A schematic illustration of the proposed oral-brain axis working model that depicts the potential relationships between periodontal inflammation, extracellular vesicles (EVs) from the saliva, systemic inflammatory signaling, blood-brain barrier (BBB) dysfunction, microglial activation, and neuroinflammatory processes. The model is hypothetical, and a salivary EV-mediated pathway from Periodontitis to neurodegeneration has yet to be established.

## Methodology

This review aimed to present a detailed summary of available evidence on salivary extracellular vesicle (EV) proteomics and the potential molecular association between periodontitis and neurodegeneration using a structured narrative approach. The systematic literature search used elements of PRISMA-2020 style reporting solely as a transparency tool, and the narrative synthesis brought together mechanistic evidence, new concepts, and translational aspects beyond what quantitative synthesis alone could provide.

### Literature Search Strategy

This study used a structured narrative review with a well-defined literature search and study-selection process. These databases PubMed/MEDLINE, Scopus, and Web of Science were searched for publications between 2009 and 2026 with the following keywords: periodontitis, salivary extracellular vesicles, proteomics, neuroinflammation, blood-brain barrier dysfunction, and neurodegeneration. This review used reference-list screening to identify additional relevant studies. This review follows PRISMA 2020 to present the identification, screening, eligibility, and inclusion process transparently; however, this review was not a formal systematic review or meta-analysis. Despite significant heterogeneity in study design, EV isolation method, proteomic platform, populations, and outcomes, we did not conduct a formal risk of bias assessment, grade the evidence, or perform quantitative synthesis. Therefore, this review interprets the included evidence through the lens of study type, evidence consistency, evidence directness, and methodological limitations.

### Study Selection

A total of 974 records were identified through PubMed/MEDLINE, Scopus, and Web of Science. 276 duplicate records were removed, and 698 records remained for screening of titles and abstracts. Of these, 506 records were excluded due to scope and predefined eligibility criteria. The remaining 192 articles were further assessed for full text, and 80 were excluded according to previously defined exclusion criteria, such as review articles, editorials, conference abstracts, non-English publications, lack of molecular evidence, and research not in the field of this review. Last, a qualitative narrative synthesis was performed on 112 studies. Figure 2 shows the full study identification and selection process.

**Figure 2.**
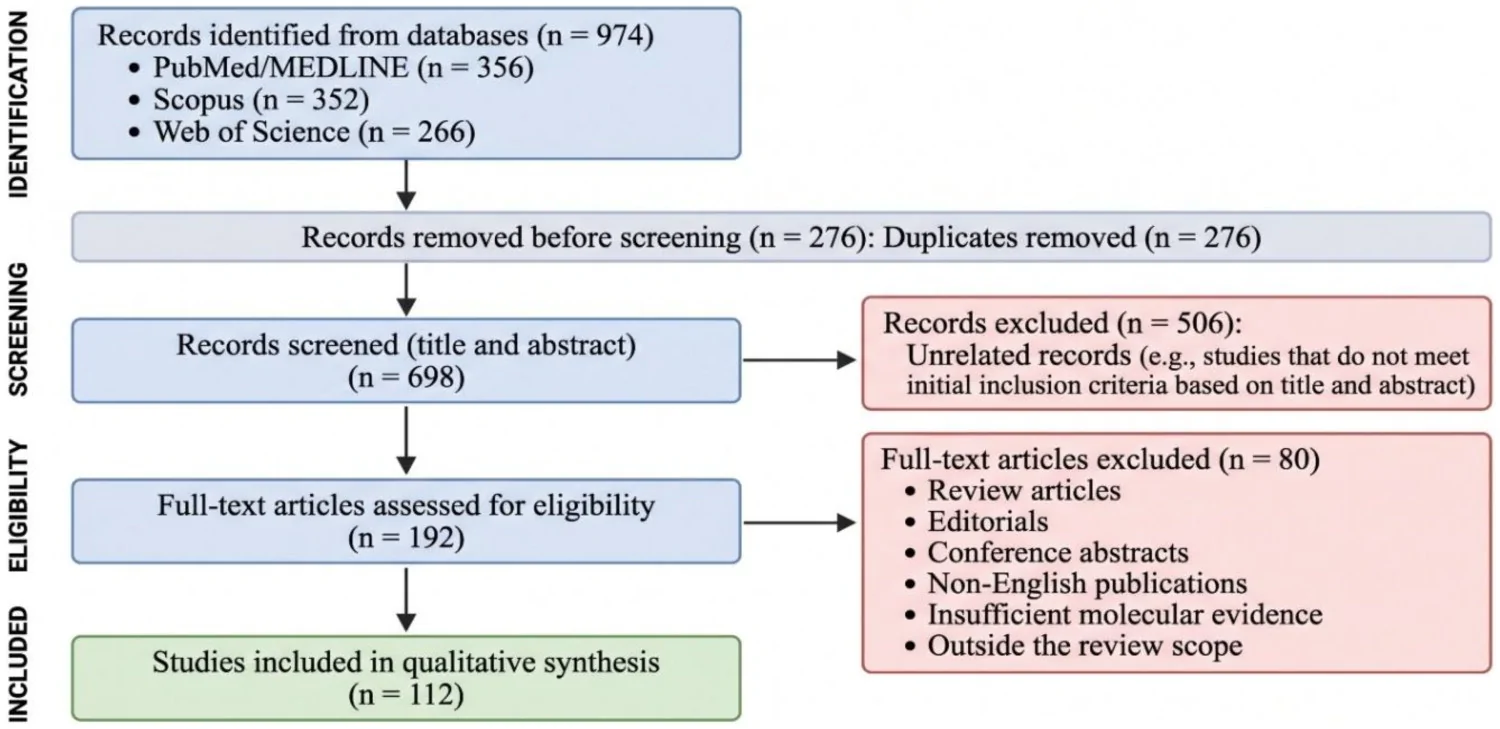
PRISMA-based workflow illustrating the literature identification, screening, eligibility assessment, and final study selection process.

### Eligibility Criteria

Eligibility criteria were original research articles that examined salivary EVs, EV proteomics, neurodegenerative diseases, blood-brain barrier changes, neuroinflammation and related molecular mechanisms, and periodontitis. Studies that reported proteomic analyses, biomarker discovery, extracellular vesicle characterization, or mechanistic investigation that are relevant to the review objectives were included. Only articles written in English were taken into account. Review articles, editorials, conference abstracts, letters to the editor, book chapters, and non-English publications were excluded. Studies with insufficient molecular evidence, studies not on extracellular vesicles of the saliva, and publications not related to periodontitis and neurodegeneration were also excluded from the final synthesis.

### Narrative Evidence Synthesis

The included studies showed significant heterogeneity in study design, sample characteristics, extracellular vesicle isolation techniques, proteomic technologies, and reported outcome measures. Evidence was narratively synthesized by compiling molecular pathways, protein signatures, biological mechanisms, and translational evidence that recurred across the documents. Areas of agreement, areas of inconsistent findings, methodological limitations, and current knowledge gaps were critically discussed to allow a balanced interpretation of the available evidence. This integrated approach provides an overview of the salivary EV proteome that may provide a mechanistic link between periodontal inflammation and neurodegenerative disease and highlights key areas for further investigation and translation to the clinic. This review’s novelty lies in its integrative approach to the salivary EV proteome in the context of periodontitis and neurodegeneration. It compiles the latest knowledge on the mechanistic pathways, proteomic signatures, biomarker potential, and EV heterogeneity, and outlines emerging fields of therapeutic engineering, single-EV technologies, and AI-driven diagnostics. These strategies are still largely future-oriented and specific to salivary EVs and will need additional validation to assess their potential for diagnosis and therapy.

Foundational studies established core concepts and mechanisms, while recent studies, especially those published between 2024 and 2026, were incorporated to capture emerging evidence and advances in the field. Results of the recent reports were interpreted with caution when the results were not replicated by other independent groups, validated in a prospective fashion, or supported by broader clinical evidence.

## Periodontitis as a Source of Systemic Molecular Signaling

### Periodontal Dysbiosis and Tissue Destruction

Periodontitis begins with a dysbiotic subgingival biofilm that triggers a chronic inflammatory response in the gingival pocket. The pocket wall is swollen, ulcerated, and filled with inflammatory cells. The epithelial barrier is no longer sealed, allowing bacterial products, host mediators, and cell debris to pass beyond the local site. Neutrophils accumulate in large numbers and act as a first line of defense, yet their excessive activation also adds to tissue injury through degranulation and protease release. Neutrophilic elastase affects epithelial adhesion and can also promote deeper bacterial penetration; the same cell type that protects tissue can also damage tissue in chronic disease (Neurath & Kesting, 2024; Pan et al., 2019). Periodontal tissue damage is driven not only by bacterial challenge but also by the host inflammatory response. The host response is a major source of destructive signaling. Pro-inflammatory cytokines such as IL-1β, TNF-α, IL-6, IL-17, and IL-23 are increased in periodontal lesions and perpetuate a cycle of immune activation, fibroblast stress, and bone loss. Complement activity also mediates this process, as C3-linked signaling can enhance dysbiosis and amplify inflammatory circuits (Martínez-García and Hernández-Lemus, 2025). In addition, matrix metalloproteinases, particularly MMP-8, MMP-9, and MMP-13, degrade collagen and other matrix components, and the structural system of the periodontium deteriorates over time. This is because periodontitis is a chronic condition that generates molecular spillover; thus, it is not only an oral condition (Martínez-García & Hernández-Lemus, 2025; Pan et al., 2019; Thantry, 2026). Figure 3 summarizes the molecular steps involved in converting periodontal inflammation into protein-rich salivary EVs, from the initial bacterial challenge to subsequent host inflammatory signaling, protein shedding, and EV cargo packaging.

**Figure 3.**
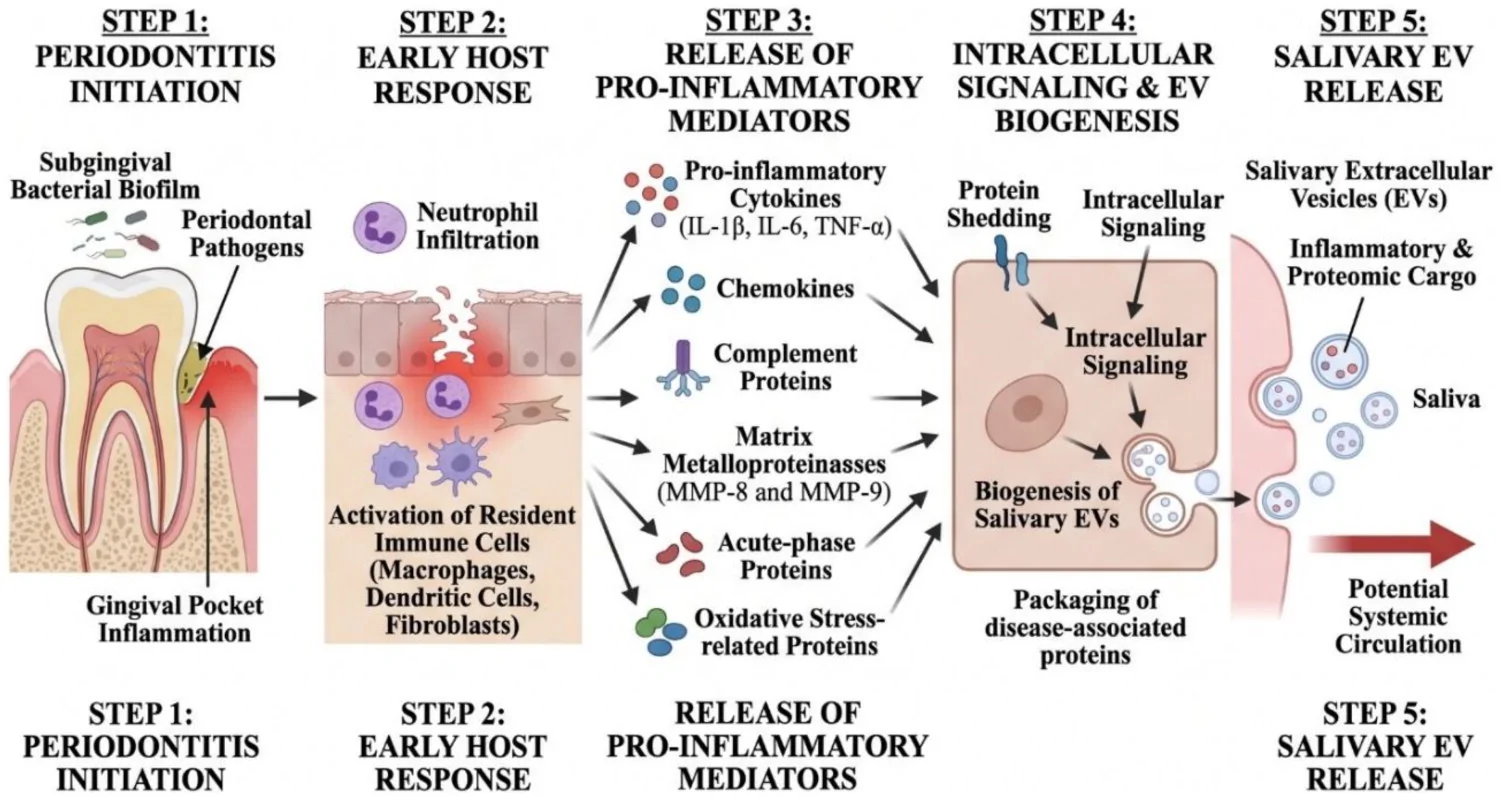
Proposed molecular cascade from periodontal inflammation to salivary extracellular vesicle (EV) alterations. Experimental and clinical evidence support periodontal inflammatory responses and EV-associated changes, while the complete sequence and its implications for systemic or neurological effects remain speculative.

### Molecular Mediators Released in Periodontitis

A wide spectrum of mediators is exuded from the inflamed periodontal lesion in gingival crevicular fluid and saliva. This profile focuses on cytokines. The cytokines IL-1β, IL-6, TNF-α, IL-17, and IL-23 promote leukocyte influx, osteoclast activation, and continued inflammatory signaling. Chemokine activity is an added dimension as it directs neutrophils, monocytes, and T cells to the lesion. These mediators also shift the gingival inflammatory response into a more systemic inflammatory response (Ramadan et al., 2020). The cytokine network in periodontitis is constantly evolving, and newer reviews now refer to this as a linked network or circuit of cytokines and not just markers (Ahmad et al., 2025; Martínez-García & Hernández-Lemus, 2025). The inflammatory response is further accompanied by acute-phase proteins associated with IL-6 signaling. Periodontitis may increase circulating C-reactive protein and fibrinogen, which indicate a more generalized acute-phase response (Polepalle et al., 2015). Dysregulated complement activity also promotes persistent tissue injury and microbial persistence, providing additional amplification. MMPs subsequently break down the extracellular matrix and release other inflammatory components. This profile is accompanied by oxidative stress-related proteins and ROS. Periodontal lesions can produce excess ROS, and some of this oxidative load may enter the circulation. This leads to a complex molecular composition containing inflammatory, proteolytic and redox-active signals (Hajishengallis, 2010; Hiyoshi et al., 2022; Polepalle et al., 2015; Thantry, 2026). Table 1 summarizes major periodontitis-associated proteins and inflammatory mediators, along with their oral sources, biological roles, and possible relevance to neurodegeneration.

**Table 1.** Key periodontitis-associated proteins and inflammatory mediators.

Molecule or protein	Source in oral tissue	Main function	Relevance to neurodegeneration	Evidence level	Studies and references
IL-1β	Gingival epithelium, macrophages, neutrophils	Strong pro-inflammatory cytokine; drives leukocyte recruitment and tissue destruction	Supports systemic inflammation and neuroinflammatory signaling linked to AD-related pathways	Clinical: Moderate-strong for elevation in periodontitis; mechanistic/indirect: Moderate for neuroinflammatory relevance	Alarcón-Sánchez et al. (2025) Salivary IL-1β is elevated in periodontitis.
TNF-α	Macrophages, neutrophils, fibroblasts	Amplifies inflammatory cascades and activates NF-κB signaling	Linked to chronic inflammation and microglial activation in oral-brain axis models	Clinical: Moderate for periodontal inflammation; animal/*in vitro*: Moderate for neuroinflammatory mechanisms; EV-specific evidence: limited	Hashim et al., (2024) Pro-inflammatory mediators in periodontitis and Alzheimer’s disease.
IL-6	Gingival tissues, immune cells, GCF	Induces acute-phase response and supports inflammatory amplification	Associated with systemic inflammatory load and cognitive risk pathways	Clinical: Moderate-strong for periodontal inflammation; clinical/observational: Moderate for neurological association; causal evidence: insufficient	Teles et al. (2024) Inflammatory biomarkers during periodontitis progression and after treatment.
Complement C3/C5a	Complement cascade in periodontal lesions, GCF	Enhances innate immune activation and dysbiosis-related inflammation	May contribute to complement-driven neuroinflammation and brain immune activation	Animal/experimental: Moderate for periodontal complement mechanisms; mechanistic/indirect: Preliminary-moderate for neuroinflammatory relevance	Hajishengallis et al. (2021) C3-targeted therapy in periodontal disease.
MMP-8	Neutrophils, gingival crevicular fluid, saliva	Collagen breakdown and connective tissue destruction	Relevant to tissue invasion, systemic spread, and possible BBB-related matrix injury	Clinical: Strong for periodontal disease association; mechanistic/indirect: Preliminary for BBB/neurodegenerative relevance	Alarcón-Sánchez et al., (2025) MMP-8 elevated in saliva of periodontitis patients.
MMP-9	Neutrophils, macrophages, periodontal pocket fluid	Gelatinase activity; extracellular matrix degradation and barrier weakening	Plausible role in BBB disruption and neurovascular stress	Clinical: Moderate-strong for periodontal association; animal/*in vitro*: Moderate for BBB-related mechanisms; salivary EV-specific: Preliminary	Alarcón-Sánchez et al., (2025) Salivary MMP-9 elevated in periodontitis.
Oxidative stress-related proteins or antioxidant enzymes	Gingival tissues, saliva, GCF	Reflect redox imbalance, protein oxidation, and local tissue injury	Oxidative stress is a shared feature of periodontitis and neurodegeneration	Clinical/biochemical: Moderate for periodontal oxidative stress; mechanistic/indirect: Moderate for shared neurodegenerative relevance; EV-specific: Preliminary	Pouliou and Piperi (2024) Oxidative stress impact in periodontitis.
CX3CL1 or CX3CR1 axis	Periodontal tissues and immune cells	Chemokine signaling and immune cell communication	Relevant to inflammatory biomarker modeling and neuroimmune communication	Clinical/observational: Preliminary-moderate; mechanistic: Preliminary for neuroimmune relevance; direct salivary EV evidence: limited	Alarcón-Sánchez et al., (2024) CX3CL1/CX3CR1 as inflammatory biomarkers.

Evidence strength indicates the nature, consistency and directness of the available evidence and is not a formal GRADE evaluation. The clinical evidence shows an association with periodontal disease, while the mechanistic evidence comes from animal and *in vitro* studies. The term "neurodegenerative relevance" should be taken as associative or biologically plausible when no direct evidence is available.

### Relevance of These Molecules for Distant Organs

These mediators may be relevant beyond gingival tissues because they can enter the systemic circulation. Periodontitis causes multiple microleakages of inflammatory mediators into the bloodstream during chewing, oral hygiene, and continual ulceration of the pocket epithelium. Low-grade bacteremia and mediator spillover lead to an extended systemic inflammatory response (Almagbol, 2025). These findings link periodontitis to systemic inflammation and high mortality risk, and large-cohort data support the concept of the oral lesion as a chronic peripheral driver of body-wide immune load. Functionally, the periodontal pocket is a chronic site of production of inflammatory mediators, some of which could travel to distant organs via the systemic circulation (Pink et al., 2023; Romandini et al., 2026).

One of the initial distant targets is the vascular endothelium. Circulating cytokines, acute-phase proteins, complement products, proteases, and oxidants can activate endothelial cells, increasing adhesion-molecule expression and impairing vascular homeostasis (Liao, 2013). This disrupts endothelial barrier function, increases local oxidative stress, and causes endothelial injury. This molecular burden can also induce neurovascular stress because cerebral microvasculature control relies on tight endothelial regulation. Over time, this stress can promote blood-brain barrier dysfunction and a pro-inflammatory brain environment (H. Wang et al., 2026). Therefore, periodontitis should not be overlooked as a source of molecular messages within EVs and a potential upstream mediator of alterations in EV cargo that could eventually signal oral-to-brain communication.

## Salivary Extracellular Vesicles: Biogenesis, Isolation, and Proteomic Landscapes

### EV Biogenesis and Types

Salivary extracellular vesicles are membrane-bound particles, small carriers that reflect the status of their cells of origin. They arise through ordered membrane traffic and store proteins in a protected form. Salivary extracellular vesicles contain a heterogeneous population from different cellular origins and biogenesis pathways. Unless an experimental determination of a particular EV subtype or biogenesis pathway has been made, the general term “extracellular vesicles (EVs)” is employed throughout this review in line with current ISEV guidelines. Small extracellular vesicles (sEVs) are used when referring to vesicles defined primarily by their physical characteristics. The term ‘exosome’ is used only for vesicles for which an endosomal origin and endosomal pathway of biogenesis has been specifically proven (Schank et al., 2026). Microvesicles are formed by budding directly off the cell surface. Apoptosis involves the appearance of apoptotic bodies, which contain larger amounts of cellular material. Although clear boundaries between these EV subtypes cannot always be established, this distinction helps design studies and report results. Existing policies on EVs emphasize the importance of clear and specific naming, marker usage, and methods, as confusing names can obscure true biological distinctions (Han et al., 2022; Théry et al., 2018).

Exosome identity should not be determined exclusively by vesicle size or general surface markers since there is an association with the endosomal sorting pathway. Cargo selection occurs via ESCRT-linked machinery and accessory pathways, after which intraluminal vesicles form; multivesicular bodies mature and are released into saliva. This pathway helps explain the protein richness of oral EVs (Gurung et al., 2021). Inflammatory stress, epithelial turnover, and immune activation may increase vesicle output. Periodontal lesions thus function as sites of EV generation rather than reservoirs. Salivary EVs at these sites may transport host proteins that reflect inflammatory and remodeling processes, as shown in EV studies of periodontitis (Han et al., 2022; M. Li et al., 2021; C. Liu et al., 2023).

### EV Origin in Saliva

Salivary EVs are not homogeneous secretions. The saliva from major salivary glands is a background of vesicles, which are being formed by the secretory cells of these glands, and many are found in whole saliva (Han et al., 2023). Gingival crevicular fluid is an additional factor, particularly in the case of periodontal inflammation (Han et al., 2022). Vesicles are shed from oral epithelial cells during normal epithelial turnover and in the event of an injury. During neutrophil influx and macrophage activation, periodontal lesions release even more vesicles filled with immune cells. Currently, researchers cannot reliably identify the exact cell type and EV subpopulation responsible for possible oral-brain communication from bulk salivary EV preparations, and no disease-specific salivary EV population has been identified as a systemic or CNS-directed carrier. This mixed origin provides salivary EVs with a strong diagnostic value, but it also poses some problems with pre-analytical control and interpretation (Dodds et al., 2015; Xulu et al., 2026).

The salivary matrix is complex and varies continuously. It contains water, ions, mucins, enzymes, antimicrobial factors, and many soluble proteins. EV transport in this fluid is important, and EVs can remain functional because the lipid bilayer prevents rapid cargo degradation. That’s important for biomarker work. Free proteins are susceptible to dilution, cleavage, and trapping in the matrix, while EV cargo frequently remains intact and amenable to enrichment and profiling. EVs produced by salivary glands enable non-invasive sampling and repeated collection over time. Saliva is also a convenient output of oral health and oral stress (de Paula et al., 2017; Dodds et al., 2015; Xulu et al., 2026). Nevertheless, definitively identifying EVs originating from salivary glands is difficult, as surface markers often do not allow unambiguous discrimination of EVs from salivary glands, gingival tissues, oral epithelial cells, immune cells, or other sources. Hence, caution must be used when interpreting the cellular origin of bulk salivary EV preparations, and the suggested involvement of any particular EV population in transmitting the oral-to-brain message is only speculative and needs to be accompanied by appropriate source- or cell-specific experimental data.

### Proteome Composition of Salivary EVs

Salivary EV proteome is wide-ranging and complex. Membrane proteins are abundant and may include tetraspanins, transport proteins, and cell-adhesion and vesicle-identity receptors. During oral disease, inflammatory proteins (related to cytokines) contribute an inflammatory signature (Teles et al., 2024). Many EV datasets in saliva contained complement proteins, acute-phase proteins, and innate immune regulators, consistent with the concept of oral EVs carrying messages that define innate immune regulation. Proteins known as chaperones help stabilize proteins and their stress response. Proteases and redox-linked proteins also emerge, along with enzymes and enzyme regulators (Niforou et al., 2014). Lipid-associated proteins and apolipoproteins are responsible for the reinforcement of the membrane and are possibly involved in cargo transport. Adhesion molecules may mark epithelial stress, immune cell interaction, and tissue remodeling. Salivary EVs have been proteomically profiled, and these cargo classes have been suggested to be different depending on disease state, method of collection, and method of isolation (Han et al., 2022; M. Li et al., 2021; Wu et al., 2023). This cargo diversity matters because it links biology to function. Membrane proteins affect uptake and targeting. Host defense is encoded in immune proteins. The presence of chaperones and enzymes indicates stress, metabolic change, and tissue repair. These proteins might be found in higher concentrations in EVs from gingival tissues and crevicular fluid in periodontitis and end up in the saliva as a molecular signature of gingival disease (Cui et al., 2024). This profile is useful for biomarker discovery and mechanistic studies. It also describes the transition away from bulk saliva proteins toward vesicle-focused proteomics because vesicle contents are often more specific than bulk saliva proteins (Han et al., 2022; M. Li et al., 2021; Xulu et al., 2026). Figure 4 lists some of the most important protein classes found in EVs in saliva, as well as their potential diagnostic and mechanistic significance.

**Figure 4.**
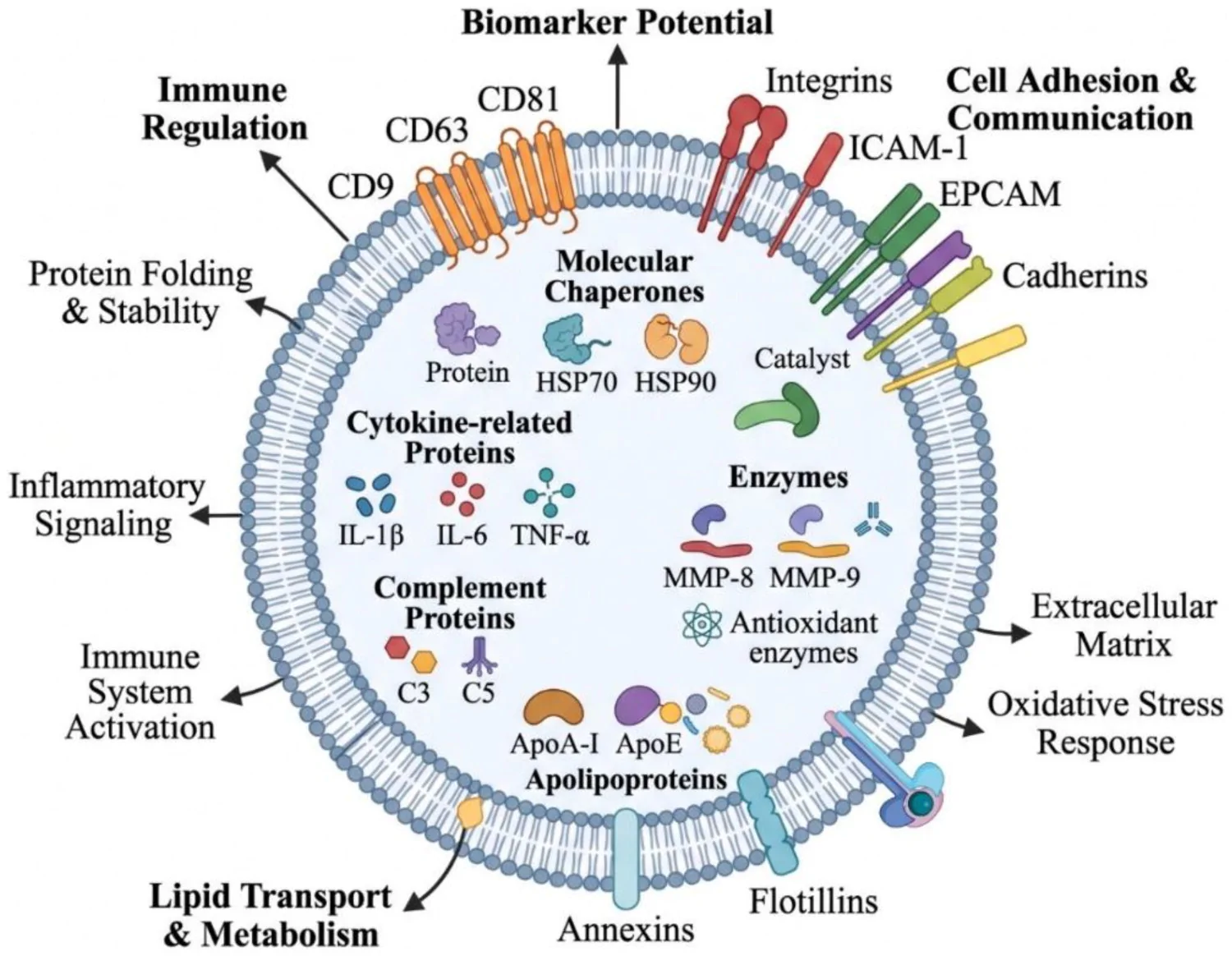
Illustration of the proteomic salivary extracellular vesicles.

### Isolation and Characterization Workflow

Pre-analytical variables include eating, drinking, oral hygiene, exposure to mouth rinse, collection time, storage temperature, and freeze-thaw cycles. These factors can affect yield and can cause downstream misinterpretation. Centrifugation removes cells and large debris from the sample. Ultracentrifugation has long been used for EV enrichment, but it can co-isolate protein aggregates and lipoproteins. Size-exclusion chromatography separation is cleaner than harsh precipitation steps, and vesicles often remain intact. Specificity is improved with immuno-capture (by targeting surface markers; however, it can be restricted in the recovered population). There is no ideal method; the selection of workflow should be based on the purpose of the research, the volume of the sample, and the requirements for purity and/or yield (Reseco et al., 2024; Sidhom et al., 2020).

Using nanoparticle tracking analysis, particle size and concentration are provided. The shape and membrane structure of vesicles are demonstrated by transmission electron microscopy. A rough hydrodynamic profile is provided via dynamic light scattering. Confirmation is typically by Western blotting of the marker proteins, typically comparing EV-enriched proteins with depleted controls. LC-MS/MS offers the most comprehensive insight into the salivary EV proteome and facilitates pathway-level interpretation. The best studies combine physical and molecular evidence, not just one assay. This aligns with current EV reporting standards and will improve comparability between laboratories. It also facilitates the shift from descriptive EV counting toward functional proteome mapping (Ogawa et al., 2024; Sidhom et al., 2020; Yu et al., 2023). Salivary extracellular vesicles are, overall, a useful crossroad between oral biology, immunology and proteomics. Their biogenesis provides insight into cargo packaging. Their oral sources indicate disease relevance. They have a protein landscape that underlies their value for diagnosis. You need to have a disciplined workflow due to their isolation. These properties combine to create a strong platform for studying oral inflammation and its broader biological implications through salivary EVs. The main techniques employed for EV isolation, purification, and characterization, along with their pros, cons, and suggested use, are presented in Table 2.

**Table 2.** Summary of EV isolation, purification, and characterization methods.

Method	Principle	Strength	Limitation	Best use	Reference
Pre-analytical control	Standardized saliva collection, storage, and handling before EV workup	Reduces noise and improves reproducibility	Oral hygiene, diet, time of day, and freeze-thaw cycles can still affect samples	All salivary EV studies	(Cui et al., 2024; Lan et al., 2025)
Differential centrifugation	Stepwise removal of cells and debris by centrifugal force	Simple and widely used	Can carry over proteins and larger particles	First-step EV enrichment	(Dilsiz, 2024; Lan et al., 2025)
Ultracentrifugation	High-speed centrifugal pelleting of vesicles	Traditional method, useful for bulk EV recovery	Time-consuming, can co-isolate aggregates and lipoproteins	General EV enrichment before downstream analysis	(Cui et al., 2024; Dilsiz, 2024)
Size exclusion chromatography	Separation by size through porous resin	Higher purity and gentler than harsh pelleting methods	Dilutes the sample and may need reconcentration	Proteomics and biomarker-focused work	(Cui et al., 2024; Lan et al., 2025)
Immuno-capture or immunoaffinity	Antibody-based binding to EV surface markers	High specificity and good host-EV enrichment	Marker-dependent and may miss EV subtypes	Targeted biomarker studies in saliva	(C. Liu et al., 2023)
Nanoparticle tracking analysis (NTA)	Tracks particle movement to estimate size and concentration	Gives particle count and size distribution	Does not confirm EV identity alone	Routine EV characterization	(Kowkabany & Bao, 2024)
Transmission electron microscopy (TEM)	Direct imaging of vesicle shape and morphology	Strong visual confirmation of vesicles	Low throughput and labor-intensive	Structural validation	(Malatesta, 2021)
Dynamic light scattering (DLS)	Measures hydrodynamic particle size in suspension	Fast and easy to run	Less accurate for mixed populations and aggregates	Supporting size analysis	(Jia et al., 2023)
Western blot	Detects EV marker proteins and negative controls	Confirms molecular identity	Marker panel can be limited, and method is semi-quantitative	EV confirmation after isolation	(Kolenc & Maličev, 2024)
LC-MS/MS	Mass spectrometry-based protein identification and profiling	Deep proteomic coverage and pathway insight	Needs clean prep and specialized analysis	Salivary EV proteomics and biomarker discovery	(Das et al., 2021)

Epithelial cells, immune cells, gingiva and salivary glands are the sources of salivary EVs. Their proteome carries inflammatory, structural, and transport proteins that reflect oral disease. Saliva is variable and mixed and must be carefully collected, enriched, and characterized. This section presents a perspective on EVs as stable, information-rich vehicles that can aid in biomarker identification and mechanistic oral-brain studies. The overall workflow for salivary EV generation, isolation, purification, characterization, and proteomic analysis is illustrated in Figure 5, highlighting the sequential steps required for reliable EV-based biomarker studies.

**Figure 5.**
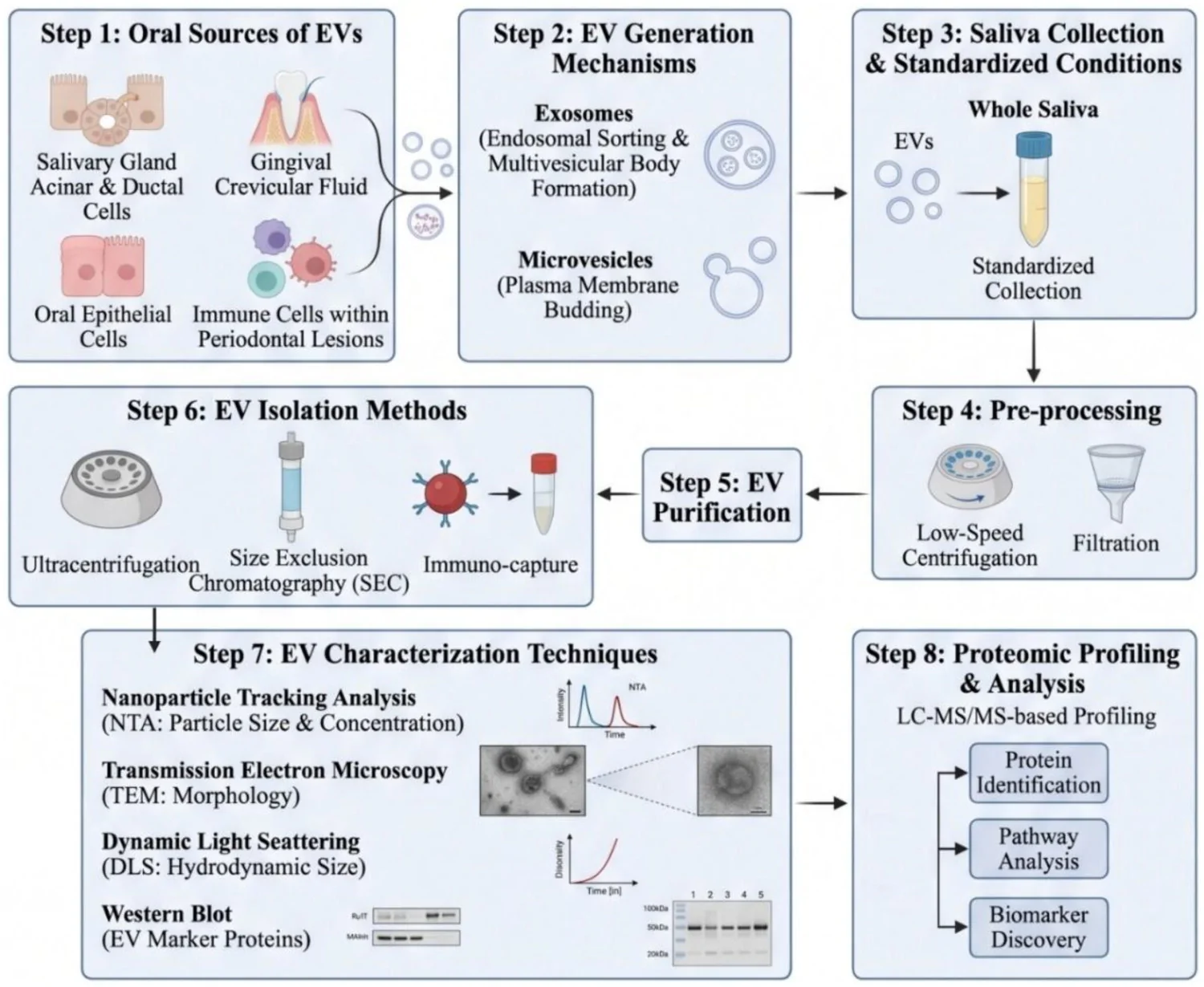
Schematic workflow of salivary EV biogenesis and isolation from oral sources to purified proteomic analysis.

## Mechanistic Link between Periodontitis and Neurodegeneration

Periodontitis is a persistent inflammatory burden that is not confined to the oral cavity (X. Li et al., 2022). Bacterial challenge, tissue injury, and host defense all generate protein signals that can enter saliva, be packaged in extracellular vesicles, and enter the blood. Such a movement provides the oral lesion with a path to other organs of the body, such as the brain. The proposed relationship is not limited to bacterial dissemination. Host proteins, inflammatory mediators, and redox stress signals seem to shoulder much of the burden. Salivary EVs enhance this model, as they help to maintain signal quality and aid in intercellular transfer across tissues, while also protecting the cargo (X. Li et al., 2022; Plachokova et al., 2024; Vega Sanchez et al., 2026).

### Systemic Dissemination of EV Proteome

Salivary EVs could be a biologically stable carrier for transporting molecular cargo, as the lipid membranes found in these EVs could shield associated molecules from degradation (Cui et al., 2024). Detecting disease-associated cargo in salivary EVs does not imply systemic dissemination or delivery to the brain. While there is evidence for EV interactions with circulation and the BBB, the evidence of transport of periodontitis-associated salivary EV populations *in vivo* is still not fully defined. Therefore, any systemic and neurological effects reported to be caused by salivary EVs should be regarded as possible steps of the potential continuous pathway from the oral cavity to the brain and not as definite. Proteins are more stable when bound to vesicles than free proteins in the saliva, as they can be diluted or degraded as they move through the oral cavity and circulation. This finding suggests that proteomic messages can be transported and that periodontal inflammation is reflected in the proteome (Wu et al., 2023). EVs derived from gingival tissues, crevicular fluid, and oral epithelial cells may contain proteins associated with cytokines, complement factors, chaperones, enzymes, and adhesion molecules, which correspond to the disease activity of the gingival tissue (Han et al., 2022). Several studies in the field of saliva EVs have demonstrated that EVs are not mere by-products of saliva. They serve as biologically active, highly informative, and diagnostic carriers with interesting mechanistic implications (Wu et al., 2023).

Although evidence shows EVs are present in circulation and other models, it is unknown whether periodontitis-associated salivary EVs enter circulation or reach the brain. An interaction between saliva and the brain has been suggested, but the specific subpopulation of EVs in saliva that mediates this interaction remains to be demonstrated experimentally. EV cargo might therefore lead to a more extensive inflammatory field associated with periodontitis than just a purely oral event. In that environment, it is possible that vesicles associated with these proteins will follow the vesicles and that these proteins may be involved in distant tissue stress, including proteins linked to innate immunity, matrix turnover, and oxidative stress. This potential pathway is a biologically plausible but not fully confirmed route and direct evidence of a direct path through the saliva from periodontitis to the nervous system still has to be established (Berumen Sánchez et al., 2021; R. Li et al., 2024; Plachokova et al., 2024).

### Blood-Brain Barrier Dysfunction

Integrity of the tight junctions, stability of endothelial cells, and regulation of transcellular transport are essential for the blood-brain barrier (Kouki et al., 2022). The balance can be upset by the presence of cytokines, complement products, proteases, and oxidative mediators found in the circulation in periodontal inflammation. Experimental studies have correlated periodontitis with decreased barrier integrity, decreased expression of claudin-5, and increased permeability of the barrier in brain tissue. The barrier then becomes more permeable to inflammatory traffic and immune activation (Ben Amara et al., 2026; Jiang et al., 2025). The brain is highly susceptible to the smallest alteration of endothelial behavior and vascular permeability (Kouki et al., 2022; R. Li et al., 2024), and that change is of paramount importance.

A major component of this process is the activation of the endothelial cells. Abnormal production of IL-6, TNF-α, and associated mediators may lead to increased adhesion molecules, disruption of redox balance, and loss of barrier control. MMP-9 is of particular significance here, as it can break down matrix components and compromise barrier structure. MMP-9 has been linked to degradation of the extracellular matrix and to barrier changes of the BBB, and experimental studies have linked periodontitis to changes in BBB integrity and endothelial stresss (Y. Hu et al., 2024). This suggests a possible mechanistic link between periodontal inflammation and neurovascular dysfunction, but the molecular mediator(s) and causative order remain unclear. Likewise, it is not yet known whether the salivary EVs from periodontitis patients carry an active component that could be involved in BBB disruption, endothelial activation, or microglial stimulation (Kwon et al., 2026; Shang et al., 2023). Figure 6 depicts one proposed model of how systemic inflammatory signaling, EV-endothelial interactions, BBB dysfunction, and neuro-inflammatory responses may be related. Notably, BBB dysfunction does not provide evidence that EVs generated from periodontitis cross the BBB, and EVs are not shown to be directly transported into the CNS.

**Figure 6.**
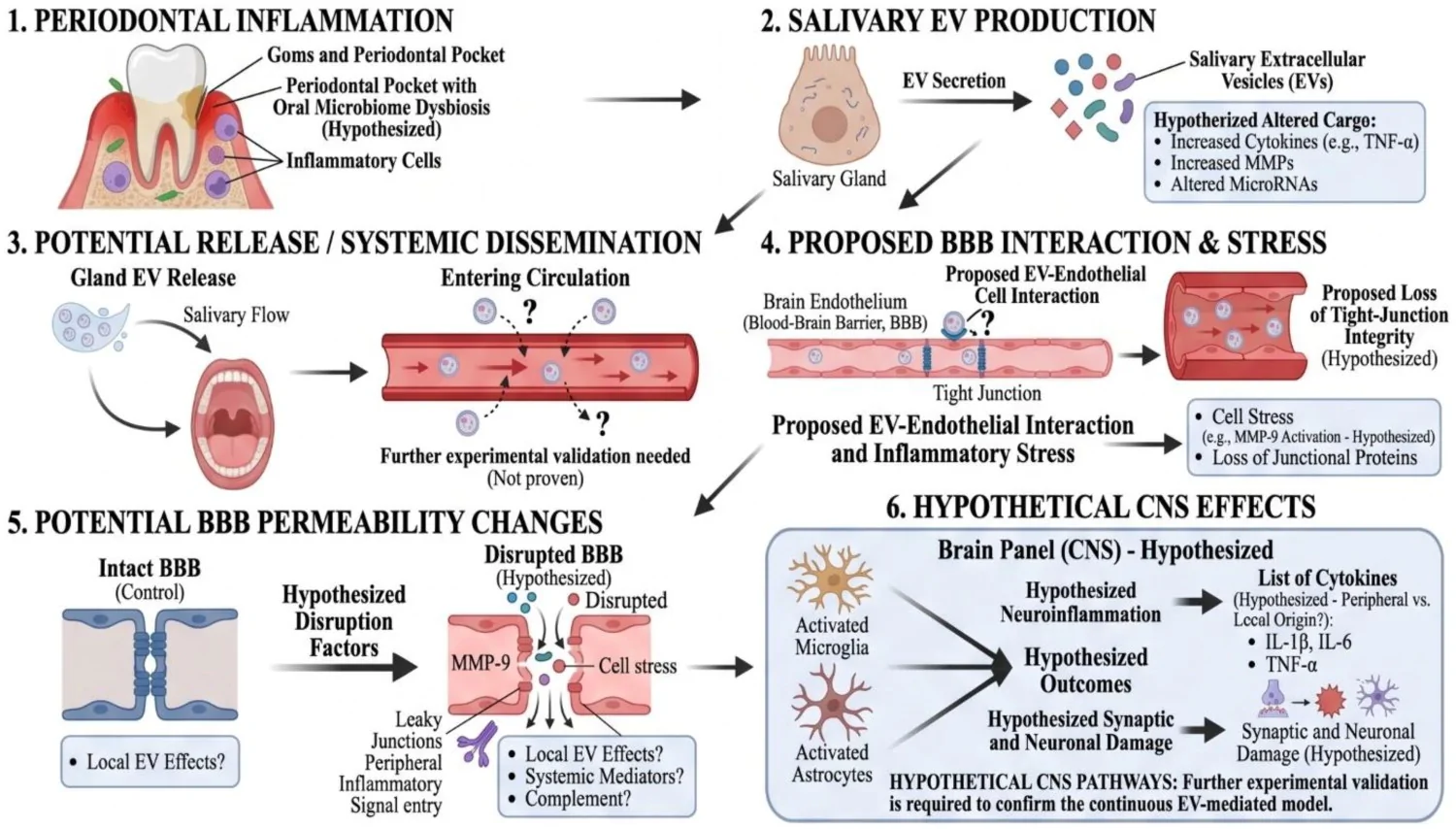
Illustrates the suggested connections between periodontal inflammation and the systemic inflammatory signaling, EV-endothelial interactions, BBB dysfunction and neuroinflammatory responses. Experimental data suggest that BBB changes are related to periodontitis, but it is not yet known whether EV-induced barrier dysfunction or salivary EV transport to the CNS occurs during periodontitis. Therefore, the sequence shown is not a proven sequence but a model.

### The Role of Microglial Activation and Neuroimmune Signaling

Microglia are resident cells of the central nervous system, which serve as first responders to danger signaling. If the barrier is compromised, peripheral inflammatory signals can prime microglia toward an activated state. Once activated in this way, microglia produce higher levels of inflammatory mediators, reactive species, and cytokines, and the response feeds upon itself. Astrocytes may also contribute to this process. It may be able to react to the same stress messages, amplify inflammation, and contribute to chronic neuroimmune tone. This is a theme found in oral-brain axis research as well as in the larger field of neuroinflammation studies (Jiang et al., 2025; Mayer & Fischer, 2024).

Periodontitis can increase the systemic inflammatory set point, and that inflammation can prime brain immune cells before significant damage occurs. This leads to chronic inflammatory amplification. Experimental evidence shows that ligature-induced periodontitis is associated with cognitive impairment, neuroinflammation, and molecular changes characteristic of AD (Kouki et al., 2022; Seyedmoalemi & Saied-Moallemi, 2025). In other periodontal pathogen models, the blood-brain barrier transitioned and microglial states shifted toward an inflammatory profile. This bridge may explain a progressive shift from oral disease to brain vulnerability, particularly with aging, through comorbidity or repeated exposure to inflammatory signals (Furutama et al., 2020; Jiang et al., 2025; Kouki et al., 2022).

### Molecular Pathways to Emphasize

Several pathways have been proposed to mechanistically connect oral inflammation with neuroinflammatory processes. Toll-like receptors (TLR2 and TLR4) sense bacteria and activate NF-κB, the master switch that regulates inflammatory cytokine transcription. This pathway is strongly associated with neuroinflammatory signaling, salivary immune cargo, and periodontal disease. The NF-κB network is one of the most evident connections between microbial stress and chronic immune activation and is upstream of numerous downstream inflammatory products (Anirudhan et al., 2020; Didilescu et al., 2024).

The NLRP3 inflammasome operates at another level. The periodontal microbiota can activate the NLRP3 sensor in oral tissue, and NLRP3 activation can lead to the release of IL-1β and IL-18. NLRP3 is also involved in neuronal injury and chronic microglial activation in neurodegenerative disease. The inflammasome has two roles: in periodontal disease progression and in brain pathology. The same applies to the HMGB1-RAGE axis. In periodontal tissue, HMGB1 functions as an alarmin, increases with disease severity, and maintains chronic inflammation through RAGE-mediated signaling. Other components of this network are complemented C3 and C1q (Didilescu et al., 2024; Hao et al., 2022). Complement activation contributes to periodontal inflammation and is well known to be involved in microglial activation and synaptic tagging in the brain (Didilescu et al., 2024; Hao et al., 2022). Figure 7 shows the major molecular network involved in the development of periodontal disease and its relationship with neurodegenerative injury, through coordinated signaling of TLRs, cytokines, complement, inflammasome activation, and BBB-related proteins.

**Figure 7.**
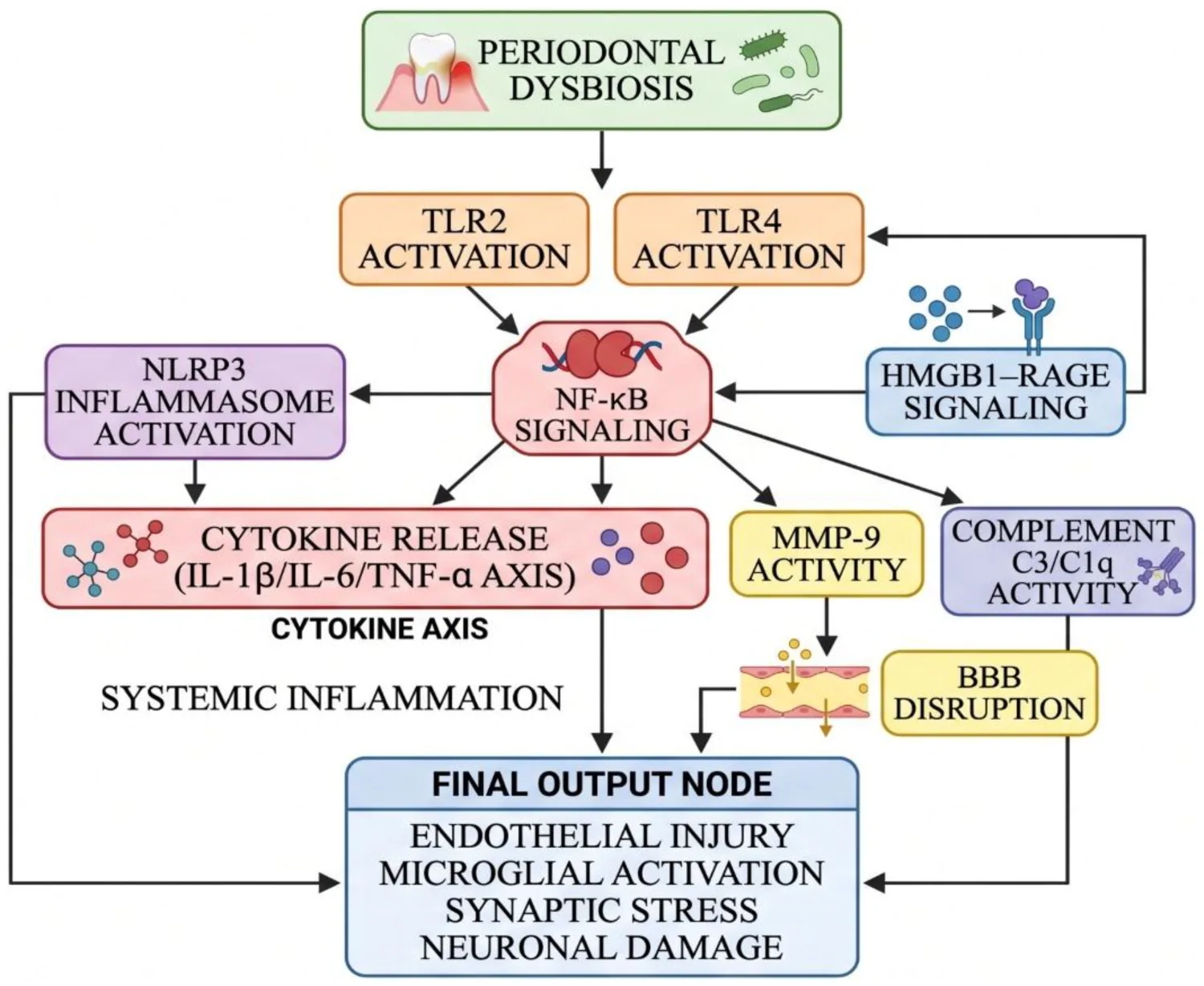
Schematic diagram of potential molecular network that connects periodontal inflammatory pathways with neuroinflammation. The integrated salivary EV-mediated pathway is hypothetical.

#### Evidence Strength and Interpretation of the Proposed Oral-Brain Pathways

In this review, findings, indirect/m Mechanistic evidence, and hypothesis-generating concepts are deliberately separated. Although clinical and experimental studies have reported associations between periodontitis, systemic inflammation, vascular dysfunction, and neuro-inflammatory changes, direct evidence that periodontitis-associated salivary EVs can transport specific molecular cargo to the brain and directly cause changes in neurodegenerative processes remains limited. The oral-brain EV pathway is therefore proposed as a biologically plausible, testable framework rather than a defined, continuous pathway.

The proposed oral-brain relationship must be understood in light of the available evidence’s type and quality. Clinical evidence primarily demonstrates the link between periodontitis and neurodegenerative outcomes, while animal and *in vitro* evidence links periodontal disease to neuroinflammation, microglial activation, and blood-brain barrier (BBB) changes. EV studies in saliva suggest alterations in EV content and cargo associated with disease, but specific evidence that EVs from periodontitis reach the brain and elicit these neurological effects remains scarce. Hence, the shared molecular pathways should not be interpreted as evidence of a confirmed EV-mediated causal pathway, but rather as a biologically plausible interface (Almarhoumi et al., 2023; Alvarenga et al., 2021; Banks et al., 2020; Mansouri et al., 2025).

Importantly, data showing that periodontitis affects systemic inflammatory or neurological processes should not be taken as evidence that salivary EVs mediate these effects. Likewise, if circulatory EVs can interact with or cross the BBB, this does not prove that EVs found in the saliva of patients with periodontitis do so. Current evidence does not, however, strongly establish a direct link between periodontal inflammation and neurodegeneration through a continuous pathway of salivary EVs. This is especially significant as EV transport through the BBB is not fully understood, and there is considerable variability from EV source and experimental model. The summary of the evidence for the proposed periodontitis-salivary EV-brain pathways and their current interpretation is summarized in Table 3.

**Table 3.** Evidence strength and interpretation of proposed pathways linking periodontitis, salivary EVs, and neurodegeneration.

Proposed mechanism	Current evidence	Evidence type	Interpretation	Refs.
Periodontitis - systemic inflammation	Moderate	Human observational studies	Association is supported; causality for neurodegeneration remains uncertain	(Alvarenga et al., 2021)
Periodontitis - microglial activation	Moderate	Animal and *in vitro* studies	Experimental evidence supports neuroinflammatory responses, but not a salivary EV-specific mechanism	(Almarhoumi et al., 2023)
Periodontitis - BBB dysfunction	Moderate	Animal studies	BBB alterations have been demonstrated experimentally; the responsible molecular mediator remains uncertain	(Jiang et al., 2025)
TLR2/TLR4-NF-κB and inflammatory signaling	Moderate	Animal/*in vitro* and disease-pathway evidence	Biologically relevant to both periodontal inflammation and neuroinflammation, but EV-specific CNS activation is unproven	(Almarhoumi et al., 2023; Mansouri et al., 2025)
NLRP3/cytokine signaling - neuroinflammation	Moderate	Experimental evidence	Supports mechanistic plausibility; direct mediation by salivary EVs remains unestablished	(Kong et al., 2025)
Salivary EV cargo - systemic/brain delivery	Preliminary	Salivary EV and EV-transport studies	Salivary EV cargo is detectable and disease-associated, but systemic dissemination and CNS delivery of specific periodontal EV populations remain to be demonstrated	(Banks et al., 2020; C. Liu et al., 2023)
Salivary EVs - BBB disruption/microglial activation	Preliminary or speculative	Indirect evidence	EVs can interact with BBB models, but this does not establish that periodontal salivary EVs produce these effects *in vivo*	(Banks et al., 2020; C. Liu et al., 2023)

The evidence discussed in this review is divided into three categories for clarity: direct evidence, indirect or mechanistic evidence, and hypothesis-generating evidence. Direct evidence: evidence from studies that specifically show a defined EV-associated process or clinical relationship. Indirect evidence consists of evidence from periodontal studies, systemic inflammatory studies, BBB studies, animal studies, and neuroinflammatory studies that suggests biological plausibility but does not provide evidence of a salivary EV-mediated pathway. Hypothesis-generating evidence suggests relationships that require experimental or longitudinal validation. As such, the periodontitis-salivary EV-brain pathway described in this review should be viewed as a working model, not an experimentally demonstrated continuous pathway.

The pathway is completed by oxidative stress and mitochondrial injury. Periodontitis increases ROS, disturbs the mitochondrial balance, and contributes to redox pressure in tissues and circulation. This burden can facilitate endothelial injury, endothelial barrier leakiness, and neuronal stress. Oxidative stress also promotes protein misfolding, lipid damage, and persistent inflammation. The various steps in the overall pathway are thus bacterial dysbiosis, activation of innate immunity, cytokine generation, inflammasome priming, complement activation, damage to the epithelial barrier, and mitochondrial stress (Shang et al., 2023). These pathways aren’t mutually exclusive. They also reciprocally support each other and participate in a tight inflammatory network (Plachokova et al., 2024). Table 4 summarizes the principal molecular pathways connecting periodontitis with neurodegenerative disease.

**Table 4.** Summary of the main oral-to-brain pathways, their oral sources, CNS effects, and disease links.

Pathway/ protein	Oral source	CNS effect	Associated neurological condition	Main mechanistic role	Refs.
TLR2/TLR4-NF-κB	Periodontal biofilm, gingival epithelium, immune cells	Microglial priming, cytokine amplification	Alzheimer’s disease, vascular cognitive impairment	Innate immune sensing and inflammatory transcription	(de Molon et al., 2026)
NLRP3 inflammasome	Gingival immune cells, inflamed periodontal tissue	IL-1β/IL-18 release, neuroinflammatory amplification	Alzheimer’s disease, broader neurodegeneration	Inflammasome-driven pyroptotic signaling	(Didilescu et al., 2024)
RAGE-HMGB1	Damaged periodontal cells, inflamed gingiva	Endothelial activation, DAMP signaling	Alzheimer’s disease	Alarmin-mediated inflammatory amplification	(Nogueira et al., 2014)
Complement C3/C1q	Periodontal inflammatory milieu, GCF, immune cells	Microglial activation, synaptic tagging, neuroinflammation	Alzheimer’s disease	Complement-driven immune pruning and inflammatory spread	(Hajishengallis, 2010; Hao et al., 2022)
MMP-9-BBB disruption	Neutrophils, periodontal pocket fluid, saliva	Tight-junction loss, BBB leakiness	Vascular cognitive impairment, Alzheimer’s disease	Matrix degradation and barrier injury	(Jiang et al., 2025; Turner & Sharp, 2016)
IL-1β / IL-6 / TNF-α axis	Gingival epithelium, macrophages, neutrophils	Neuroinflammation, glial activation, endothelial stress	Alzheimer’s disease, Parkinson’s disease	Systemic cytokine propagation and brain immune activation	(de Molon et al., 2026)
Oxidative stress and mitochondrial injury	Inflamed periodontal tissue, activated neutrophils	Neuronal stress, redox imbalance, barrier injury	Alzheimer’s disease, Parkinson’s disease, vascular cognitive impairment	Redox-mediated tissue damage and chronic inflammatory persistence	(Z. Li et al., 2025; Pouliou & Piperi, 2024; Shang et al., 2023)

### Disease-Specific Relevance

#### Alzheimer’s Disease

The clinical and experimental evidence linking periodontitis and Alzheimer’s disease is in its infancy and, largely associative and mechanistic, supports the association. Experimental investigations have found alterations in the BBB, immune system priming, and AD-associated pathology after periodontal challenge. Salivary EVs could mirror these inflammatory changes associated with the disease, but the causal role of salivary EVs in Alzheimer’s disease remains undefined. The most definite and consistent association with periodontal disease is Alzheimer’s disease. Clinical studies show a significant correlation, and experimental studies show barrier dysfunction, immune priming, and AD-like pathology following periodontal challenge (X. Li et al., 2022; R. Li et al., 2024). Changes in the salivary EV proteome in this context could reflect the inflammatory changes seen in the disease, but a causal relationship between these changes and neurodegenerative processes remains undetermined. Microglial activation, the complement pathway, and persistent cytokines fit well with the amyloid and tau models of disease progression. Thus, the oral-brain axis provides a viable opportunity to better comprehend part of the inflammatory burden observed in Alzheimer’s disease (Kouki et al., 2022; R. Li et al., 2024; Seyedmoalemi & Saied-Moallemi, 2025).

#### Parkinson’s Disease

Evidence linking periodontitis and Parkinson’s disease is relatively sparse, with findings pointing to possible links to systemic inflammation, oxidative stress, and immune activation that may increase vulnerability of dopaminergic pathways; however, these observations are mostly associative or mechanistic, and no salivary EV-mediated pathway has been identified. Oral inflammation is relevant, and Parkinson’s disease doesn’t seem to be as closely associated as Alzheimer’s disease. Under chronic periodontal stress, there is potential for systemic inflammation, oxidative damage, and immune activation (priming), favoring increased vulnerability of the dopaminergic system. The amount of evidence here is less, but the biological logic is consistent (Bui et al., 2019). Vascular cognitive impairment is also well positioned, because endothelial injury, BBB leakage, and chronic inflammatory signaling fit this phenotype well. There may be more than one mechanistic field in which many overlapping themes exist, such as barrier dysfunction and immune dysfunction, which also occur in other diseases, such as multiple sclerosis, and broader dementia syndromes. The main point is that periodontitis does not require a specific brain target to develop. It can create a general inflammatory terrain which increases the risk for a number of neurodegenerative conditions (Bui et al., 2019; Plachokova et al., 2024; Zohud et al., 2026).

In summary, inflammation, alterations in vascular barrier function, and periodontitis-related neuroimmune signaling may be associated with neurodegenerative mechanisms through several biologic pathways. Pathways involved in both periodontal and neuroinflammatory studies include TLR2/TLR4–NF-κB, NLRP3 inflammasome, HMGB1-RAGE, complement C3/C1q, MMP-9 and IL-1β/IL-6/TNF-α signaling, and oxidative stress. However, these common pathways do not prove that salivary EVs from periodontitis necessarily trigger them. Although salivary EVs may offer a quantifiable platform for studying disease-associated molecular signals, the direct relevance of EVs in linking periodontal inflammation to neurodegeneration needs experimental proof.

## Salivary EV Proteome as a Biomarker Platform

The salivary EV proteome is very suitable for biomarker work, as saliva is easy to collect, repeatable, and low burden for patients. The signal is often cleaner than free saliva proteins, as EVs protect their protein cargo from rapid degradation. Recent reviews and method studies have validated salivary EVs as a stable readout of oral disease and systemic conditions. This enables their use in screening, follow-up, and multi-marker testing over time.

### Salivary EV Proteome Is Biomarker-Friendly

Salivary EVs are appealing because they are non-invasive and easy to sample. Repeated collection does not require needles, trained venipuncture personnel, or high patient tolerance. This provides saliva with a very practical advantage for long-term monitoring. Saliva also reflects not only local oral conditions but also systemic immune stress, neurodegenerative changes, and periodontal inflammation, all of which can be captured in the same sample. EV-enriched fractions are more stable and less enzymatically damaged than whole saliva, as the membrane of EVs prevents enzymatic degradation. This argument is reiterated in recent reviews and isolation of EVs in saliva (Cui et al., 2024; Han et al., 2022; Théry et al., 2018). Both periodontitis and neurodegeneration are dynamic, meaning that a biomarker platform should be robust to repeated sampling and provide a consistent response. Salivary EV proteomics is more suitable than invasive tissue sampling. It can also be a better fit than single free-protein assays, as EV cargo may convey a more comprehensive and stable molecular state (Corana et al., 2025). Recent studies have identified markers for periodontitis that can change over the course of the disease and following treatment, which encourages the use of saliva as a tool to monitor disease progression and not just make one-time diagnoses (Alavi et al., 2025; Corana et al., 2025; Teles et al., 2024)

### Candidate Biomarker Categories

The salivary EV proteome can be used to identify multiple biomarker groups. The first class is inflammatory proteins, such as IL-1β, IL-6, TNF-α, IFN-γ, IL-8, and MMP-8, which have been detected in saliva during periodontal disease and its response to treatment (Corana et al., 2025). Complement proteins complement this by reflecting chronic inflammatory stress and tissue damage. Saliva-related proteins play a role in the oral-brain axis, particularly in biomarker studies of neurodegenerative diseases, where lactoferrin, tau, amyloid-β, and α-synuclein have been studied repeatedly (Dumitru et al., 2026; Jiao et al., 2024). These proteins may be matrix-remodeling proteins, endothelial stress markers (including MMP-9), and adhesion-linked proteins related to the BBB. Oxidative stress markers and chaperone proteins are also important, as redox imbalance and protein handling are similar across periodontal inflammation and brain disease (Świątek et al., 2026). This list is not a set panel. It serves as a virtual pool of candidates. These candidates should therefore be considered discovery-stage or hypothesis-generating markers, not clinically validated markers. Requires testing of clinical utility in adequately powered, independent cohorts; testing of diagnostic performance such as sensitivity, specificity, and AUC; testing for analytical and biological reproducibility; and prospective longitudinal testing. Single proteins may not be very effective biomarkers, and the optimum systems will most likely be combinations. Salivary protein panels have previously been shown to separate disease states more clearly in periodontitis studies. The same is true for neurodegenerative saliva research. Reviews of the salivary biomarkers in Alzheimer’s disease and in Parkinson’s disease indicate that individual markers are unreliable, and combinations may be more relevant for clinical application (Alavi et al., 2025; Jiao et al., 2024; Teles et al., 2024).

### Diagnostic Process

The first step in any practical diagnostic workflow is standardized saliva collection. Results vary with time of day, eating habits, oral hygiene, smoking, storage temperature, and the number of freeze-thaw cycles. Pre-analytical control is thus as important as the assay. EV enrichment is performed after collection through centrifugation, ultracentrifugation, size exclusion chromatography, or immuno-capture. Each method has its own advantages and disadvantages in yield, purity, and cost. More recent method studies and EV guidelines emphasize that no single method will fit all studies. Sample volume, downstream assay platform, and research purpose should dictate the method used (Cui et al., 2024; Mohamed et al., 2012; Théry et al., 2018). Proteomic profiling may be carried out after enrichment either by LC-MS/MS for discovery or by targeted assays for validation. Discovery work can be used to establish cargo patterns and pathway shifts, and ELISA or targeted mass spectrometry can be used to evaluate the most promising candidates in larger sample sets (Boulestreau et al., 2024; Ogata et al., 2022). Researchers then use several proteins to create a multi-marker score that provides a single clinical readout. That score needs to be correlated with periodontal stage, systemic status, and the cognitive/neurological phenotype before it can become meaningful. These kinds of progressive research steps from wide discovery to narrow validation are supported by salivary biomarker studies in periodontitis and neurodegeneration (Alavi et al., 2025; Corana et al., 2025; H. Hu & Leung, 2023; Ogata et al., 2022; Teles et al., 2024).

### Strengths and Weaknesses of Biomarker Translation

Salivary EV Proteome work is non-invasive, repeatable, and appropriate for longitudinal monitoring. It also provides access to immune, proteolytic, and structural proteins, as well as stress-related proteins, in one fluid. The vulnerabilities are just as tangible. When biomarkers are low abundance, sensitivity may drop. Oral inflammation, systemic disease, and medication effects can overlap, reducing specificity. Collection and isolation need to be carefully controlled; otherwise, reproducibility can be low. Evidence is also inconsistent, with some studies identifying strong candidates and others indicating small or insignificant effects (Boulestreau et al., 2024; Cui et al., 2024; Teles et al., 2024). Salivary protein output is altered by age, smoking, diabetes, oral hygiene, periodontal therapy, and a number of medications (Alavi et al., 2025; Jiao et al., 2024). The platform itself is not impaired by this reality; it requires discipline. Translating biomarkers in this area will require standardization of collection, unambiguous EV isolation, appropriate controls, and validation in larger cohorts. However, the salivary EV proteome shows great promise as a biomarker platform, with the ultimate clinical step being reproducible, biologically sensible, outcome-based panels.

Biomarker discovery is separate from clinical validation. A disease-associated EV protein cannot be considered a clinically useful biomarker unless it is repeatable in new populations and on different analytical platforms, with clinically relevant diagnostic value. Going forward, future studies should move from discovery cohorts to independently validated cohorts and, eventually, prospective longitudinal studies. Before salivary EV signatures are used routinely in the clinic, researchers must establish clinical thresholds and investigate sensitivity, specificity, AUC, reproducibility, and treatment-related changes (Zhuang et al., 2026).

## Therapeutic Opportunities and Translational Engineering

Inflammation in periodontitis is often chronic, and this can become a therapeutic opportunity. Salivary EVs are a natural carrier system that is membrane-protected, biologically compatible, and has a strong oral origin. These vesicles can be engineered to become a delivery device when the disease signal is present.

### Salivary EVs as Therapeutic Carriers

Salivary EVs are appealing since they align with the body’s own transport system. Their lipid bilayer helps to protect cargo from enzymatic attack and helps them to persist in biological fluids under harsh conditions. They also originate in the mouth, allowing non-invasive collection and study. Salivary EVs have been reported as stable diagnostic units and as potential therapeutic vectors, as they can be used to transport proteins, nucleic acids, and small molecules with good structural protection (Cui et al., 2024; Dhal et al., 2026). Biocompatibility is an advantage; EVs are liposomal in nature and hence are less recognized by the immune system than many synthetic nanoparticles. This feature allows reuse and reduces the chance of rapid clearance. Low immunogenicity is also important with the oral and systemic applications, particularly in chronic inflammatory diseases (Xia et al., 2024). More recent EV immunogenicity studies caution that EVs can also be recognized, and that this depends on the source, cargo, and surface characteristics; hence, it cannot be taken for granted. The practical point is simple. EVs are friendly carriers, not magical ones (Bhom et al., 2025; Witwer, 2024; Xia et al., 2024). The next step is targeting potential; EV surface proteins can affect tissue tropism and uptake. This enables direct targeting of vesicles to inflamed tissue, endothelial cells, or neural targets through membrane decoration or ligand display. The EVs in saliva can also be exploited as a locally available system for oral delivery concepts, as oral biofluid systems are already used for therapeutic thinking and disease monitoring. This renders them an obvious link between the science of biomarkers and the design of drug delivery (Cui et al., 2024; Du et al., 2025; Q. Liu et al., 2023).

### EV Engineering Strategies

Engineering enhances the value of native EVs. Cargo can be loaded by incubation, electroporation, sonication, extrusion, transfection, freeze-thaw, or endogenous loading during vesicle generation. Each route varies in loading efficiency, vesicle integrity, and scalability. Membrane modification has been an important approach to improve targeting, tissue delivery, and circulation behavior of engineered EVs, as reported in a review of engineered EVs (Q. Liu et al., 2023; Meng et al., 2026). That design logic is based on surface modification. To enhance selective binding, researchers can attach peptides, antibodies, aptamers, and receptor-binding motifs to the vesicle surface. A hybrid vesicle design is also available, in which EVs are integrated with synthetic liposomes or membrane fragments to enjoy the natural recognition properties of EVs and the controlled manufacture of synthetic vesicles. Synthetic mimics also offer other benefits and provide greater batch control. Although not the ultimate solution, these hybrid systems provide an intermediate solution between biology and engineering (Du et al., 2025; Q. Liu et al., 2023). The loading with anti-inflammatory proteins is particularly relevant for periodontal/oral-brain studies. A carrier that mimics biological signaling could carry a therapeutic cargo to reduce inflammation, redox stress, and/or barrier injury. This also applies to RNA regulators. Small RNAs can silence inflammatory nodes, reduce cytokine production, and influence the local immune environment. The concept is that of "modular design". The vesicle is the shell and the payload is the active part (Du et al., 2025; S. Li et al., 2024; Q. Liu et al., 2023; Meng et al., 2026; Welsh et al., 2024). Figure 8 summarizes the main engineering routes for salivary EVs, from native vesicle collection to therapeutic payload delivery and target engagement.

**Figure 8.**
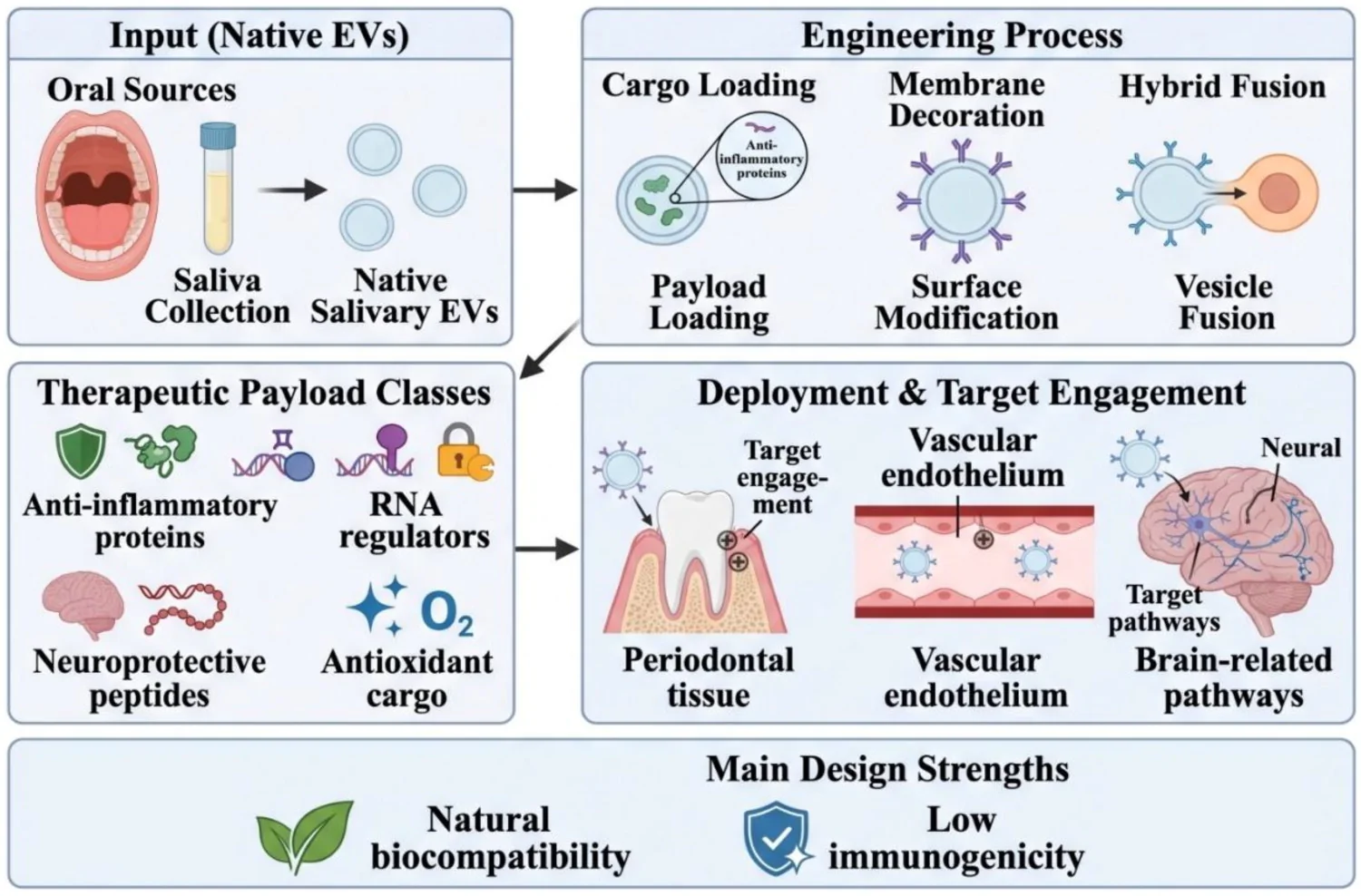
Schematic illustration of therapeutic engineering of salivary extracellular vesicles through cargo loading, surface modification, targeted delivery, and translational therapeutic applications.

### Therapeutic Payloads of Interest

The most relevant payload classes are anti-inflammatory proteins, RNA regulators, enzyme inhibitors, neuroprotective peptides, and antioxidant-associated proteins. Anti-inflammatory proteins can reduce the oral burden of cytokines and help maintain a more balanced systemic environment. RNA regulators can inhibit nodes like NF-κB, NLRP3 or any upstream inflammatory drivers. Proteases that destroy tissue and barrier integrity can be targets for enzyme inhibitors. Peptides with neuroprotective properties can provide a brain-oriented perspective, as they can help neurons survive or reduce excessive glial activation (S. Li et al., 2024; Q. Liu et al., 2023; Meng et al., 2026; Welsh et al., 2024). Proteins associated with antioxidants should also be considered. These redox imbalance, mitochondrial stress, and inflammatory amplification are common features in both periodontitis and neurodegeneration. A single antioxidant delivery vesicle can break a number of destructive cycles simultaneously. Work in saliva already supports the notion of salivary EVs as carriers of disease-related proteins and as potential therapeutic carriers in oral and systemic diseases. There is strong translational promise, but payload selection matters for disease stage and target tissue. If one does not match the context very carefully, a blunt anti-inflammatory tool can miss the real biology (Cui et al., 2024; Du et al., 2025; S. Li et al., 2024). Table 5 summarizes the main EV engineering strategies and their translational strengths and limitations (Q. Liu et al., 2023; Meng et al., 2026; Piffoux et al., 2021; Wen et al., 2025; Zeng et al., 2023).

**Table 5.** Therapeutic strategies based on EV engineering and clinical translation potential.

Strategy	Cargo or target	Intended effect	Advantage	Limitation	Refs.
Direct cargo loading	Anti-inflammatory proteins	Reduce local and systemic inflammatory burden	Simple concept, biologically aligned, strong anti-inflammatory potential	Loading efficiency and cargo retention can vary	(Piffoux et al., 2021)
RNA loading	miRNA, siRNA, mRNA regulators	Silence inflammatory pathways and reprogram immune signaling	Strong mechanistic control over target genes	Stability, delivery efficiency, and off-target effects remain concerns	(Zeng et al., 2023)
Surface modification	EV membrane proteins, peptides, antibodies, aptamers	Improve tissue targeting and uptake	Better selectivity and delivery precision	Surface engineering can alter vesicle behavior	(Q. Liu et al., 2023)
Hybrid vesicle design	EV + liposome or synthetic membrane fusion	Combine natural EV function with controllable formulation	Better engineering flexibility and scalable design potential	Fusion complexity and product consistency can be difficult	(Meng et al., 2026)
Synthetic EV mimics	EV-like nanocarriers	Reproduce EV delivery features with higher batch control	Easier manufacturing and standardization	May lose native EV biology and cell-recognition features	(Meng et al., 2026; Wen et al., 2025)
Process standardization	Identity, purity, potency, release criteria	Support reproducible clinical-grade product development	Essential for regulatory acceptance and translation	Demands strict quality control and harmonized protocols	(Wen et al., 2025)

### Clinical Feasibility

Stability, scalability, regulatory clarity, and manufacturing standardization are paramount for clinical feasibility. Stability matters because the vesicle must maintain its structure and cargo during storage, transport, and administration. Scalability is important as a good lab method is not enough to use in patients. Regulatory issues are related to product identity, release requirements, purity, potency, and batch-to-batch consistency. In other words, EV work must be transparently reported, use validated methods, and include clear quality control before clinical use, as stated in MISEV2023 and echoed in recent translation reviews (Mohak & Fabian, 2025; Welsh et al., 2024; Witwer, 2024). The final product depends on the source tissue, culture conditions, isolation pathway, and cargo loading. That means a therapeutic EV is not just a vesicle. A specified biological product and special process requirements. Hence, translational reviews repeatedly highlight the key barriers of comparability, reproducibility, and scale-up. Collecting EVs from saliva is convenient, and EVs are conceptually linked to oral disease. However, clinical use will require harmonized protocols, potency testing, and outcome-linked validation in human cohorts (Bhom et al., 2025; Cui et al., 2024; S. Li et al., 2024).

Collectively, salivary EVs could shift from passive biomarkers to active therapeutic platforms. They are useful for inflammatory disease control, oral repair, and potential delivery of neuroprotective agents due to their natural membrane structure, low immunogenic profile, and engineering flexibility. The path from concept to clinic is always tough, but the concept, natural carrier, engineered cargo, controlled manufacture, and outcome-based validation are now a workable framework in the field. The real translational route is that path, and it is already evident. Figure 9 summarizes the translation pathway from laboratory studies of salivary EVs to clinical application of salivary EVs as therapeutics.

**Figure 9.**
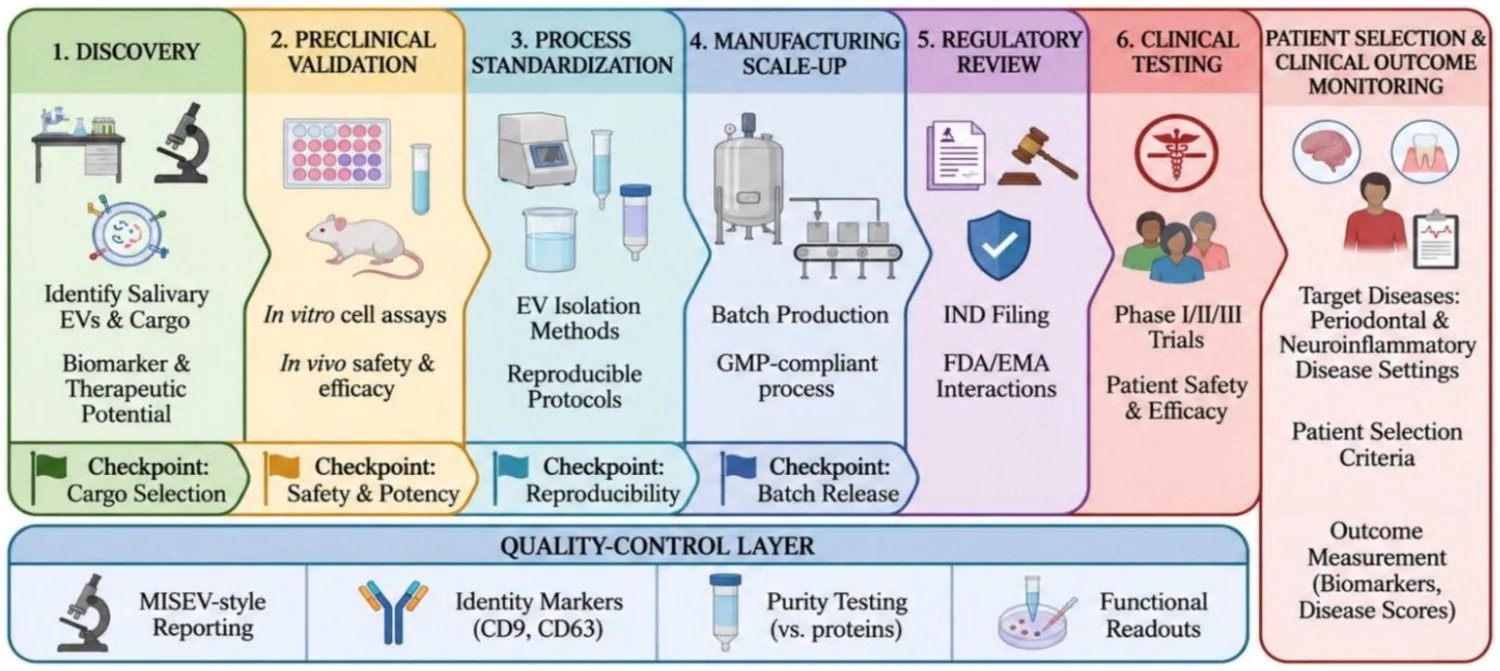
Schematic roadmap for salivary extracellular vesicle therapeutics from discovery to clinical validation and implementation.

## Salivary EV Proteome Heterogeneity and Disease-Specific Cargo Signatures

Salivary extracellular vesicles are not a single uniform group. These make up a mixed population that is distinct in size, membrane markers, cell origin, and contents. This diversity matters because bulk analysis can smooth out the underlying biology into a mean signal. EV heterogeneity has become an established characteristic of vesicle studies, and in other recent reviews, the focus has been on the fact that population-level measurements do not reflect the function and disease signals of subtypes (Bu et al., 2026; Carney et al., 2025; Théry et al., 2018). Saliva is given extra weight in this context as it is a window into the glands, gingiva, epithelial renewal, immune function, and microbial burden in a single location (Cui et al., 2024; Han et al., 2021).

Although growing evidence supports the proposed link, certain limitations and inconsistencies warrant consideration. Results from EV analysis can vary because isolation and characterization techniques differ; salivary proteomic profiles can also be affected by pre-analytical and analytical variability. Moreover, saliva-EV composition is not uniform, and different definitions of periodontitis, severity, sampling conditions, and study populations can increase variability in observed results (Boulestreau et al., 2024). Importantly, epidemiological evidence for association between periodontitis and neurodegenerative diseases is variable, and most of the evidence currently available suggests association but not necessarily causation. These factors make it difficult to compare results across studies and must be considered when interpreting the proposed oral-brain connection via EVs (Alvarenga et al., 2021). Table 6 summarizes key methodological limitations, sources of heterogeneity, and issues that must be addressed before interpreting the available evidence.

**Table 6.** Key limitations, heterogeneity, and unresolved issues in the current evidence.

Issue	Potential impact on findings	Current interpretation	Refs.
EV isolation and characterization	Different isolation and characterization procedures may yield different EV populations and cargo profiles	Limits reproducibility and cross-study comparison	(Sidhom et al., 2020)
Salivary proteomic variability	Differences in sample preparation, analytical platforms, and normalization can influence detected protein profiles	May contribute to inconsistent biomarker findings	(Alavi et al., 2025; Corana et al., 2025)
EV population heterogeneity	Bulk EV preparations may contain heterogeneous vesicle populations with different cellular origins and cargo	Limits source-specific and mechanistic interpretation	(Bu et al., 2026; Carney et al., 2025)
Periodontitis-related clinical heterogeneity	The inflammatory and EV-associated profiles can vary among patients depending on disease type and severity, comorbidities, and patient characteristics.	Differences between study populations may contribute to apparently inconsistent molecular findings and limit direct comparison	(X. Li et al., 2022; Plachokova et al., 2024)
Periodontitis-neurodegeneration evidence	There is more evidence available for Alzheimer's disease than for Parkinson's disease, and much of the literature available is associative or mechanistic, and not longitudinal or causal.	The association/biological plausibility interpretation should be applied to the relationship.	(X. Li et al., 2022; R. P. H. Wang et al., 2019)

Collectively, these limitations indicate that apparently consistent biological signals should be interpreted cautiously. Differences in EV isolation, sample handling, proteome analysis platforms, disease classification, and study populations can affect reported results and make cross-study comparisons difficult. Furthermore, epidemiological evidence is largely observational, so it does not allow definite conclusions about cause. To date, the literature supports biological plausibility, but the evidence is not sufficient to support a reproducible, causal conclusion about a role for EVs in periodontal disease and neurodegeneration.

The oral cavity adds another layer of complexity. Salivary EVs originate from the salivary glands, gingival crevicular fluid, oral epithelial cells, and immune cells within periodontal lesions. Proteomic fingerprints can vary by source. EV markers have already been shown to vary across studies on periodontitis. Others reported that a CD9-positive subpopulation was more prevalent in periodontitis, but that CD9 and CD81 levels decreased in the saliva of disease groups (Tobón-Arroyave et al., 2019). EV marker expression and inflammatory cargo profile were also enriched in immunoaffinity-enriched salivary EVs as compared to size-exclusion fractions: increased levels of IL-6, IL-8, and decreased levels of IL-10, with changes in the levels of TNF-α and osteopontin-linked signals (Han et al., 2021, 2023; C. Liu et al., 2023; Tobón-Arroyave et al., 2019). These findings strongly indicate that periodontitis does not simply increase overall vesicle numbers. It not only changes the shape of the vesicles, but also the vesicle pool itself.

Subpopulation-specific cargo sorting supports this. The vesicles can be separated by size-based heterogeneity, which separates vesicles with different protein loads and membrane compositions. It is possible to distinguish gland-derived vesicles from inflammatory vesicles released in periodontal lesions based on the cellular heterogeneity of the vesicles. Periodontal lesion-specific enrichment may increase the relative abundance of immune proteins, complement proteins, matrix turnover proteins, and stress markers. This profile can shift from active to chronic disease through inflammation-dependent proteome remodeling. More recently, several reviews on EV heterogeneity showed that this heterogeneity occurs in all body fluids and that the single-vesicle approach provides tools to visualize heterogeneity directly, not indirectly, as earlier work (Bu et al., 2026; Carney et al., 2025; Tobón-Arroyave et al., 2019). The significance of this change in saliva is straightforward. A marker may appear weak in bulk saliva but be strong in one disease-relevant vesicle subgroup.

The gold medal is in the disease-specific proteomic fingerprints. There may be different EV profiles for mild vs severe periodontitis. Inflammatory lesions could be more cytokine- and protease-rich than chronic low-grade lesions. Oral comorbidity may further modify the cargo, particularly with the presence of diabetes, smoking, or poor oral hygiene. These and other factors may change inflammatory burden and EV release profiles, and a particular patient population may exhibit different EV signatures under other biological stresses (Carney et al., 2025; Chiabotto et al., 2019; Cui et al., 2024; Han et al., 2021; Théry et al., 2018). The same holds true for the search for a treatment or cure for neurodegenerative diseases. The subtle vesicle signal could be hidden in the noise of whole saliva in early stages of neurodegenerative change and may be stronger and more obvious later in the disease. This idea is still in its infancy, but is considered a viable course of action in the EV field (Carney et al., 2025; Théry et al., 2018).

The new molecular value in this section is specificity. In generic saliva studies, there is typically a lot of "noise" from a mixture of many sources and many disease states. Salivary EV proteome studies can improve by separating EV subgroups and associating each subgroup with a function and a source. This strategy can serve to uncouple oral-only inflammation from oral-to-brain signaling. It can also help provide a more precise indication of disease status, activity, and response to treatment. This is already indicated by differences in the abundance of EV markers and inflammatory cargo, and in the content of DNA and RNA found in the periodontitis literature (Han et al., 2021; 2023; C. Liu et al., 2023; Tobón-Arroyave et al., 2019).

This makes salivary EV heterogeneity a valuable link between biomarker science and mechanism. Actively injured periodontium might be represented by a vesicle subpopulation containing inflammatory proteins. A second subpopulation could have matrix or adhesion signals associated with tissue remodeling. A third group of proteins could be neuroimmune-related and better suited to oral-to-brain communication. Surface markers and single-vesicle analysis platforms will be important in the future of this field, as will subtype-aware methods. Those tools can help to distinguish signal from background, and take salivary EV proteomics beyond a broad-based description to real biological precision (Boulestreau et al., 2024; Théry et al., 2018).

## Single-EV Proteomics, Spatial Biology, and AI-Driven Precision Oral-Brain Diagnostics

Single-EV analysis enables characterization of individual vesicles rather than relying solely on population-level measurements. Bulk EV data provide an average view. This average can obscure important and rare subgroups of vesicles with deep biological significance (Tran et al., 2025). Nevertheless, that change is important in saliva, as it is a mixture of vesicles from oral glands, gingiva, epithelial cells, and immune cells. A small but relevant vesicle population associated with the disease process may be drowned out by the fluid’s background noise. As of late, heterogeneity is no longer considered a nuisance in EV guidance and review work, but rather a fundamental aspect and a direct route toward enhanced biomarker resolution and biological interpretation (Manolopoulos et al., 2025; Welsh et al., 2024). Single-EV technology has now been applied to several practical approaches. Calibration, pre-analytical control and data reporting are focal points for the reliable use of single-EV flow cytometry and have been highlighted in the recently published compendium by the EV community (Welsh et al., 2023). Nano-flow approaches detect smaller particles, while imaging-based systems provide morphology and surface information. Aptamer-based capture can separate vesicle classes before readout. High-resolution techniques, single-vesicle RNA or protein assays, and multiplex phenotyping now enable investigation of vesicles at much higher resolution than bulk assays. This shift from bulk measurement to single-vesicle analysis has been regarded as a significant leap in the clinical diagnostics literature, both in terms of earlier disease detection and improved classification (Tiwari et al., 2026; Tran et al., 2025).

In spatial biology, a second layer of value emerges. Spatial proteomics maintains protein data in its spatial, tissue, and neighborhood contexts. This matters for studying vesicles because cargo information is not just a list of proteins; it also records the state of the cell and the microenvironment in which the vesicles are situated. Multiplexed super-resolution techniques are ushering in a new era in spatial proteomics, with this approach potentially being very useful to EV research to link vesicle contents to disease context and tissue of origin (Strack, 2024). In the oral part of the brain, this means a vesicle isn’t defined only by its protein list; it’s also defined by the biological context in which it formed. This is convenient for excluding background signals from the glands, as well as inflammation-related periodontal signals, and for focusing on disease-relevant cargo, excluding broad saliva signal noise (Manolopoulos et al., 2025; Strack, 2024; Tran et al., 2025).

Now, AI and machine learning enable a new level of interpretation. Single-EV datasets are rich, noisy, and high-dimensional, and cannot be easily interpreted by visual inspection. Machine learning can help with feature selection, pattern recognition, multimarker panel construction, and risk scoring. This space is divided into imaging, omics, cytometry, and clinical output, and the practical problem common to all four categories is that model quality depends on data quality, batch control, and clear external validation (Huang et al., 2026). Rule-based filtering, machine learning, and deep learning are also suggested approaches to reduce a large list of candidate biomarkers to those that are easy to assay, biologically plausible, and clinically useful in computational frameworks for EV biomarker discovery (J. Kim et al., 2026). Similarly, EV reviews in the field of neurology demonstrate their utility as vehicles for predictive, diagnostic, prognostic, and treatment response biomarker studies in brain disease (Manolopoulos et al., 2025).

Single-EV profiling may support early detection, patient categorization, treatment-response monitoring, and oral-systemic risk mapping. Rare inflammatory vesicles can signal an active tissue insult in a periodontal context, before a more general saliva assay changes significantly. In a neurodegenerative context, the same rationale can distinguish early weak signals from background variation and may improve clinical predictions. No single marker or method is the best future model. Clean single-EV capture, spatial context, AI-based pattern learning, and validation in independent cohorts are a linked system. Such a design will propel the field of salivary EV research from descriptive biology to useful diagnostics for mouth-brain (J. Kim et al., 2026; Strack, 2024; Tran et al., 2025).

## Limitations, Standardization and Directions for Future Research

Salivary EV proteome research has real promise, but the field still carries practical and biological noise. EVs are diverse, saliva is variable, oral inflammation is dynamic, and neurodegeneration takes place over years. This combination makes it scientifically interesting and translationally challenging. In fact, it’s not about being more enthusiastic. The next step requires more rigorous approaches, more precise definitions, and more robust validation across cohorts and platforms.

### Biological and Analytical Challenges

The first challenge is the heterogeneity of EVs. Salivary EVs vary in size, membrane composition, and the type of cells they are shed from and the contents of the EVs, so bulk measurements can result in an important signal being hidden in an average readout. Heterogeneity is not a second-order effect, as recent EV work reveals. It is a basic property that can assist diagnostics only if measured with care. Single-particle methods have made this abundantly clear, and the same holds for saliva, where vesicles from the glands, gingival tissue, epithelial tissue, and immune system are all found in the same fluid (Bu et al., 2026; Carney et al., 2025; Welsh et al., 2023). The second major problem is preanalytical variation. Collection time, food, oral hygiene status, hydration status, smoking, and storage conditions affect the saliva. These factors can change EV yield and cargo composition, and if collection is not standardized, reproducibility becomes fragile. The proteome can also be reshaped by inter-individual variation in age, sex, medications, oral health status, and systemic disease. A biomarker study that omits these variables can still yield results, but they may not extrapolate beyond the studied sample set (Cheng, 2024; Welsh et al., 2024). The oral microbiome confounding is important, too. Periodontitis is not only a host inflammatory disorder, but it’s also a multigenic disease. It is related to changes in the microorganisms, remodeling, and interaction of the host and pathogen. EV cargo in saliva can thus reflect host biology and microbial pressure. The overlap can be handy, but also confusing. A similar situation applies to systemic diseases, such as diabetes, cardiovascular diseases, and chronic inflammatory states, which can influence saliva and EV profiles in parallel with periodontitis. This is now recognized as a bidirectional (oral-brain) biological pathway, and with good reason (Ahmed et al., 2025; Soiniemi et al., 2024).

### Need for Standardization

Standardization is necessary to translate research on salivary EVs into clinical applications. Factors before sample analysis may affect EV yield and cargo, such as collection time, fasting, recent food intake, oral hygiene, hydration, smoking, storage conditions, and freeze-thaw cycles. Therefore, standardized protocols for collecting, processing, storing, and enriching EVs from saliva are needed to reduce technical variation (Sidhom et al., 2020). Each purification method (ultracentrifugation, size-exclusion chromatography, and immuno-capture) recovers a different subset of vesicles and yields different levels of purity, yield, and bias. Both MISEV2023 and the single-EV flow cytometry compendium highlight the importance of reporting the source, isolation, calibration, and controls of experiments, in addition to the list of markers used for the final analysis (Welsh et al., 2023, 2024). Likewise, variations in sample preparation, digestion, mass-spectrometry parameters, database searching, and normalization may reduce reproducibility between laboratories and proteomic systems.

Inter-individual variability is another barrier to translation. The generalizability of proposed biomarker signatures may be influenced by saliva composition and its relationship to age, sex, smoking, medications, oral health status, systemic diseases, and periodontal disease severity (Alavi et al., 2025; Mohamed et al., 2012; Welsh et al., 2024). Therefore, biomarker development must involve well-defined, well-documented study populations, suitable controls, clearly designed analytic workflows, and independent replication cohorts. Most importantly, candidate biomarkers from discovery studies must be evaluated independently by others, including their diagnostic performance (sensitivity, specificity, area under the receiver operating characteristic (ROC) curve), reproducibility, and clinically relevant decision thresholds, before adoption as clinically validated biomarkers. To date, no validated clinical cut-off values exist for salivary EV proteomic markers of the risk of periodontitis-associated neurodegenerative disease (Alavi et al., 2025; Ogata et al., 2022; Zhuang et al., 2026). Prospective and longitudinal studies are thus needed to determine whether EV signatures can predict disease development, differentiate clinically important disease stages, and vary over time with periodontal disease progression and/or response to therapy, as well as neurological disease progression and/or response to therapy. Standardization of the proteomics workflow is also important. Different answers for the same saliva sample will be obtained if a lab changes its preparation, digestion, mass spectrometry settings, database search, or normalization steps (Boulestreau et al., 2024; Ogata et al., 2022). This is not the only problem with saliva, but the matrix’s crowded and variable nature magnifies it. Reporting should include sample type, donor, isolation route, protein recovery, instrument settings, and validation strategy. EV translation reviews also focus on the same practical issues. Without improved quality control, batch criteria, and cross-study comparability, clinical progress will be slow (Bu et al., 2026; Carney et al., 2025; Cheng, 2024; Welsh et al., 2024).

Other hurdles for therapeutic applications include reproducible selection of EV sources, cargo characterization, isolation and purification, consistent production, potency assessment, safety evaluation, and batch-to-batch quality control. Salivary EVs, therefore, are a promising platform for research, but further clinical translation of the technology will require methodological harmonization, analytical validation, independent prospective studies, and demonstration of consistent clinical benefit

### Causality versus Association

A bigger question is about causality and association. Many of the current studies are cross-sectional, meaning that they illustrate an association between periodontitis, EV cargo, and neurological outcomes, but not direction. A weak association may still have biological value, but it does not, by itself, resolve the mechanism. Longitudinal cohorts are needed to determine whether EV signatures increase before cognitive decline, change after periodontal therapy, or reflect disease progression over time. Such evidence would propel the field to move beyond correlation to predictive medicine (M.-Y. Kim & Pang, 2025; Soiniemi et al., 2024). However, mechanistic models are still needed. Cellular systems and animal studies can determine whether periodontal inflammation changes BBB behavior, microglial state, or neuroinflammatory signaling via vesicle cargo. The oral-brain axis literature has already established a plausible route from chronic oral inflammation to brain immune activation, and the same holds for EV-mediated signaling (Ahmed et al., 2025; Tsai & Liao, 2026). Organoid systems and engineered barrier models can provide an additional translational link, given their ability to enable controlled testing of vesicle uptake, barrier permeability, and inflammatory response. Even if a biomarker panel has no mechanistic basis, it could appear tempting, but it will be susceptible to over-interpretation (Bu et al., 2026; Cheng, 2024).

### Future Directions

Future directions point to a multi-layered approach. The most important aspect will be multi-omics integration, as proteomics alone may not fully represent the oral-brain signal. EV studies increasingly incorporate proteins alongside RNA, lipids, and metabolites, which should improve disease specificity. Machine learning biomarker panels can facilitate high-dimensional data sorting, marker pattern discovery, and risk scoring. The main principle is not to look for a software solution to biology. The idea is to use computation to provide clinically useful combinations and filter noise (Bu et al., 2026; Cheng, 2024). Another obvious subsequent step is single-EV analysis. Rare but crucial vesicle classes are not captured by population averages, and recent single-particle reviews show that this heterogeneity is both a hindrance and an asset. Single-vesicle readouts could enhance the characterization of EV subpopulations associated with disease and may be used in combination with AI to better analyze these EVs in the future, but their clinical use in salivary EV research is yet to be proven (Carney et al., 2025; Welsh et al., 2023). The same applies to therapeutic EVs, as a known vesicle type with a defined cargo profile may be a better carrier than a random bulk preparation. Now, EVs are included in the field of precision medicine, and there is great interest in biomarker-based stratification and engineered delivery systems (Cheng, 2024; Tsai & Liao, 2026). Single-EV proteomics, spatial biology, and AI-based approaches should therefore be considered emerging research strategies and future directions rather than established translational or clinical applications.

The last message is clear. Until now, research on the salivary proteome and EVs has been a discipline that may not translate to clinical applications. The field’s future will depend on improved collection, more effective isolation, stronger validation, longer follow-up, and integrated analytics. Science is already solid enough to warrant the effort. The job now is to make the signal steadier than the noise.

## Conclusion

This review further corroborates the notion that EVs in saliva are not just a by-product of oral disease but are biologically active entities that may provide a plausible link between periodontitis and neurodegeneration. This evidence indicates that periodontal disease can create a proteomic and inflammatory load with a greater abundance of cytokines, complement factors, proteases, oxidative-stress mediators, and barrier-disrupting signals, which could be present in the salivary cargo of EVs. Research on periodontal inflammation and neurodegenerative diseases reports many common molecules, including TLR2/TLR4–NF-κB, the NLRP3 inflammasome, HMGB1-RAGE, complement C3/C1q, MMP-9-associated barrier alterations, IL-1β/IL-6/TNF-α signaling, and oxidative stress. While these common pathways support biochemical plausibility, they do not show that EVs from periodontitis directly activate these pathways in the brain or mediate the relationship between the two diseases. Therefore, salivary EVs should be considered a potentially important platform for exploring disease-associated molecular signals, not a proven mechanistic link. These pathways offer a unified framework for understanding how chronic periodontal inflammation may lead to endothelial dysfunction, microglial activation, and overall neurodegenerative vulnerability. Salivary EV Proteomics is appealing from a translational perspective as it is non-invasive, repeatable, and may be more stable than free salivary proteins. Despite this, key challenges remain, particularly EV heterogeneity, pre-analytical variation, differences in isolation methods, and a lack of large-scale clinical validation. Several challenges must be overcome to transition salivary EVs from promising biomarkers to clinical tools. Future research should focus on standardized, validated EV panels (multi-marker) and collection/isolation methods, as well as engineered EV strategies involving single-EV analysis and AI-based diagnostics. Once the field reaches this level, it can progress to the next, quieter, stronger signals.
